# Cardiovascular disease in idiopathic pulmonary fibrosis: a systematic review and meta-analysis of observational studies

**DOI:** 10.3389/fmed.2025.1653435

**Published:** 2025-09-22

**Authors:** Yang Li, Weili Tan, Yangyini Zhang, Fang Cao, Zhisong Wu, Yang Jiao, Jie Niu

**Affiliations:** ^1^Graduate School, Beijing University of Chinese Medicine, Beijing, China; ^2^Department of Geriatrics, Dongfang Hospital, Beijing University of Chinese Medicine, Beijing, China; ^3^Department of Intensive Care Unit, Dongfang Hospital, Beijing University of Chinese Medicine, Beijing, China; ^4^Department of Respiratory, Dongfang Hospital, Beijing University of Chinese Medicine, Beijing, China

**Keywords:** idiopathic pulmonary fibrosis, cardiovascular disease, ischemic heart disease, thromboembolic disease, pulmonary hypertension, meta-analysis

## Abstract

**Background:**

Cardiovascular (CV) comorbidities can affect drug tolerability and health outcomes in patients with idiopathic pulmonary fibrosis (IPF). This systematic review and meta-analysis aimed to quantify the magnitudes of association between IPF and both overall and specific categories of CV disease.

**Methods:**

The PRISMA guidelines and PICO model were followed. We searched PubMed, Embase, Web of Science, Cochrane Library, and Chinese Biomedical Literature Service System (Sinomed) from inception to April 2025 for studies investigating CV disease in IPF patients. Study quality was assessed using the Newcastle-Ottawa Scale (NOS). Pooled odds ratio (OR) for case-control/cross-sectional datasets and relative risk (RR) for cohort datasets were calculated using Review Manager 5.4. The *I*^2^ test was used to evaluate the heterogeneity and the sources of heterogeneity were explored through sensitivity analyses, meta-regression, and subgroup analyses. The funnel plot and Egger’s test were used to evaluate publication bias.

**Results:**

A total of 28 studies comprising 29 case-control/cross-sectional datasets and four cohort datasets were included, which indicated a positive association between IPF and CV disease (OR 2.44, 95% CI 1.84–3.24, *P* < 0.001; RR 1.44, 95% CI 1.07–1.92, *P* = 0.02). Meta-regression and maximized subgroup analyses confirmed the influence of control characteristics (*P* < 0.001), data source (*P* = 0.027), Newcastle-Ottawa Scale (NOS) score (*P* = 0.022), certainty of CV disease diagnosis (*P* = 0.027), body mass index (BMI), smoking status, and diabetes prevalence on both heterogeneity and risk estimates in the case-control/cross-sectional datasets. The OR varied across the CV disease category, with 1.14- to 2.51-fold increased risks for ischemic heart disease, thromboembolic disease, pulmonary hypertension, and other forms of heart disease.

**Conclusion:**

Idiopathic pulmonary fibrosis is significantly associated with CV disease, emphasizing the urgent need for systematic screening and risk reduction strategies in IPF patients.

**Systematic review registration:**

https://www.crd.york.ac.uk/PROSPERO/view/CRD420251013917, identifier CRD420251013917.

## 1 Introduction

Idiopathic pulmonary fibrosis (IPF) is a progressive and fatal pulmonary disease, with average survival being similar to or even worse than in many cancers (1). Pirfenidone and nintedanib have been approved for treating IPF; however, this only represents the first step in its treatment (2). IPF has a predilection for older individuals (3). In this clinical context, the disease is often further complicated by the presence of comorbidities. Torrisi et al. (4) demonstrated that incorporating comorbidities into the clinical prediction model enhances the possibility of predicting survival potential in IPF. Therefore, the IPF clinical management pathway emphasizes the evaluation and treatment of existing comorbidities, representing an additional domain for clinical intervention (5) to improve survival outcomes.

Both cardiovascular (CV) disease and IPF share a number of risk factors and most commonly affect a similar patient demographic: men over the age of 60 years with a history of smoking (6). Consequently, IPF patients exhibit a high burden of CV disease. Co-management by pulmonologists and cardiologists can benefit more IPF patients, resulting in growing research interest in the correlation between these two diseases. Evidence indicates that the presence of CV disease in IPF patients is associated with a number of negative outcomes. For example, in a retrospective cohort study of IPF patients listed for lung transplantation (7), pulmonary hypertension (PH) was significantly associated with increased mortality, regardless of severity. Although respiratory failure is the most frequent cause of death in IPF patients, CV disease is still responsible for up to 10% of deaths (8). Given the impact of CV disease on IPF, CV therapies such as statins may have dual benefits, as evidenced by observational and retrospective studies (9–11).

It is critical for clinicians to understand the extent of CV comorbidities in IPF patients. However, the magnitude of the increased CV risk in IPF has not been formally quantified in existing studies. We thus conducted a comprehensive systematic review and meta-analysis of existing observational studies to assess the association between IPF and CV risk.

## 2 Materials and methods

### 2.1 Study registration

This systematic review and meta-analysis have been conducted following the PRISMA 2020 guidelines (Preferred Reporting Items for Systematic Reviews and Meta-Analyses) (12) (Supplementary Material 1). The protocol was prospectively registered with PROSPERO (International Prospective Register of Systematic Reviews) under registration number CRD420251013917.

### 2.2 Search strategy

Two investigators (WT and YZ) independently and systematically searched five databases [PubMed, Embase, Web of Science, Cochrane Library, and Chinese Biomedical Literature Service System (Sinomed)] from inception to April 2025 for eligible studies. The key questions were formulated using the PICO method as follows: (1) P (population): IPF patients; (2) I (intervention): None; (3) C (comparison): Non-IPF controls; (4) O (outcome): CV disease. The following medical subject headings (MeSH) and free text terms were used in combination: (“idiopathic pulmonary fibrosis” OR “pulmonary fibrosis”) AND (“cardiovascular diseases” OR “myocardial ischemia” OR “ischemic heart disease” OR “pulmonary hypertension” OR “thromboembolic disease” OR “heart valve diseases” OR “heart failure” OR “arrhythmia”). Supplementary Material 2 provides the complete search strategy. The search was limited to human studies, including original articles in any language. Additionally, we manually searched the references of relevant meta-analyses and reviews to ensure no literature was missed.

### 2.3 Study selection

The inclusion criteria were as follows: (1) population-based observational studies (cohort, case-control, or cross-sectional) examining the association between IPF and CV disease risk. (2) The case group was diagnosed with IPF using valid and objective methods. The preferred definition of IPF was based on available official clinical practice guidelines. Other accepted definitions included clinically International Classification of Diseases (ICD) diagnostic codes, physician diagnosis, and medical chart review. (3) At least one CV disease outcome was reported (see Supplementary Material 3 for detailed definitions and criteria for determining whether CV disease is definite). (4) Non-IPF subjects were identified as controls. (5) The risk was quantified by extracting the total number of cases and the appropriate odds ratio (OR), relative risk (RR), or hazard ratio (HR), and 95% confidence interval (95% CI). The exclusion criteria were as follows: (1) interventional studies, expert opinions, reviews, case reports, conference abstracts, comments, or editorials. (2) Duplicate publications from the same database (only the most comprehensive version was selected).

### 2.4 Data extraction and quality assessment

Two investigators (WT and YZ) independently screened eligible studies according to the inclusion/exclusion criteria and performed duplicate data extraction with cross-verification for: first author, publication year, country, study design, sample size, control group characteristics, IPF diagnostic criteria, CV disease ascertainment methods, and CV disease types. Baseline information including sex, age, smoking status, BMI, and chronic comorbidities (e.g., diabetes), which may predispose to CV disease, were collected as covariates. CV diseases were categorized based on the ICD system (Supplementary Material 4). The Newcastle–Ottawa Scale (NOS) was used to evaluate the quality of the studies, which mainly included three domains (study selection, comparability, and outcome). Studies scoring ≥ 5 stars (out of nine possible) were considered high-quality. Any differences were resolved by consensus or by third-party opinions (YL).

### 2.5 Statistical analysis

Meta-analysis was performed using Review Manager 5.4 and Stata 17.0 software. OR and RR with 95% CI were used as the effect measure. OR represented the case-control study/cross-sectional dataset, and RR represented the cohort dataset. The Cochran’s Q test and *I*^2^ statistics were applied to evaluate heterogeneity. A fixed-effect model was selected when heterogeneity was non-significant (*P* ≥ 0.05 with *I*^2^ < 50%). Otherwise, a random-effect model was employed, followed by sensitivity analyses, meta-regression, and subgroup analyses to explore potential sources of substantial heterogeneity and evaluate result stability. Covariates including control characteristics, data sources, NOS scores, and certainty of CV disease diagnosis were considered potential sources of bias. Egger’s test and a visual inspection of the funnel plots were employed to assess publication bias.

## 3 Results

### 3.1 Study selection and characteristics

**FIGURE 1:**
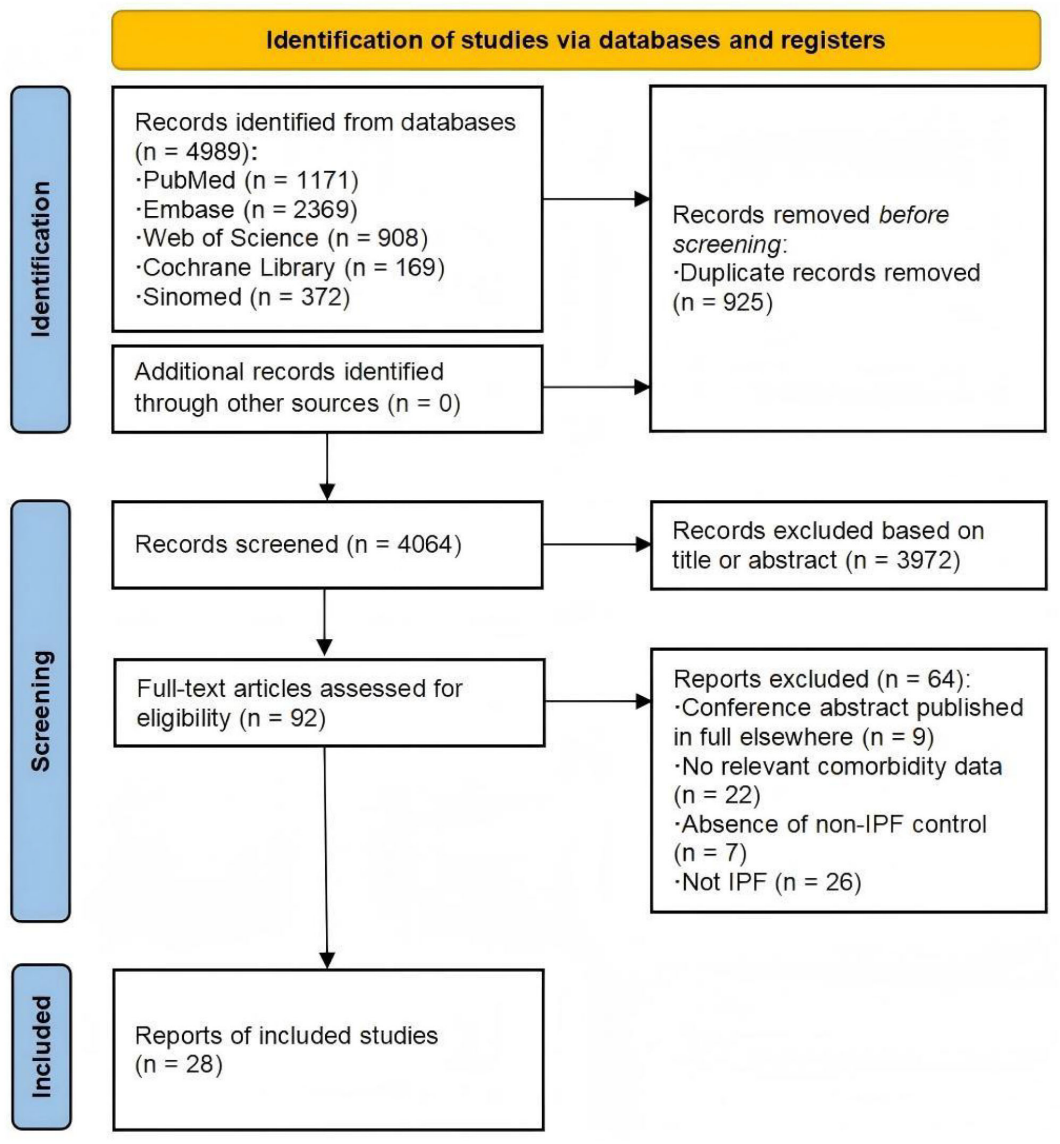
Flow diagram of study search and selection. Numbers refer to unique records, not datasets, except where otherwise indicated.

As illustrated in Figure 1, of the 4,989 related studies identified through the search, 92 underwent full-text assessment. Among these, 28 studies (13–40) met the inclusion criteria and were rated as high-quality by the NOS (Supplementary Material 5), with 13 being multicenter studies and the remaining 15 conducted in single centers. Our meta-analysis included 33 datasets from 10 countries, comprising 29 case-control/cross-sectional datasets and four cohort datasets (with four articles containing both cohort and cross-sectional components, and one article contributing two case-control datasets). In 11 studies, the IPF-non-ILD population served as controls. 15 datasets contained only the matched healthy control group, while the remaining seven datasets included patients with other chronic pulmonary diseases. IPF was diagnosed according to contemporary guidelines, with outcome ascertainment methods varying across studies and including both objective measures (e.g., laboratory tests) and subjective self-reporting. More details are displayed in Table 1.

**TABLE 1:** Characteristics of the included observational studies.

Study	Country	Data source	IG	CG	Method of IPF diagnosis	CV outcome ascertainment	CV types	NOS
Liu et al. (13)	China	MC	36	25 patients with pulmonary fibrosis as the initial manifestation of polyangiitis	ATS/ERS criteria (68)	Chart review	PH	6
Cuirong et al. (14)	China	SC	93	313 patients with ILD-non-IPF	ATS/ERS/JRS/ALAT criteria (69)	Chart review	CAD; PH; Arrhythmia	6
Bade et al. (15)	United States	MC	181	104,107 patients with lung cancer	Validated diagnostic code	Validated ICD-9 from the CHUP database	CAD; PTE; PH	6
Bray et al. (16)	United States	MC	244	244 COPD patients matched by FEV1 and 244 lifetime non-smoking control subjects matched by age.	2018 Clinical Practice Guideline Summary for Clinicians (70)	Physician diagnosis	CAD	7
Canıvar et al. (17)	Istanbul	SC	44	34 patients with SSc-ILD	The consensus of the clinicians, pathologists, and radiologists	Chart review	HF; IHD	6
Chan et al. (18)	United States	MC	99	63 patients with CTD-ILD	Validated diagnostic code	Chart review and laboratory test	IHD; AF; PH	6
Clarson et al. (19)	United Kingdom	MC	7120	56,884 control subjects matched by age and gender	Validated ICD-10	Validated ICD-10	IHD (MI)	7
Dalleywater et al. (20)	United Kingdom	MC	3211	12,307 control subjects matched by age and gender	Validated diagnostic code from THIN database	Self-report	IHD	6
Fisher et al. (21)	Canada	MC	317	428 patients with CTD-ILD	ATS/ERS/JRS/ALAT criteria (69)	Chart review	MI	6
García et al. (22)	United States	SC	100	263 control subjects matched by age, gender and residence	ATS/ERS criteria (71)	Self-report	Unspecified CV diseases	7
Hubbard et al. (23)	United Kingdom	MC	920	3593 control subjects matched by age, gender and community	Validated diagnostic code from THIN database	Chart review	IHD (acute); AF; DVT	8
Khor et al. (24)	Canada	MC	1061	2825 patients with ILD-non-IPF	Validated diagnostic code from CARE-PF database	Self-report	Unspecified CV diseases	5
Kilpeläinen et al. (25)	Finland	MC	110	127 ILD-non-IPF patients and 93 PPF patients	Chart review	Turku University Hospital data lake	Unspecified CV diseases	5
Kato et al. (26)	Japan	SC	568	790 patients with ILD-non-IPF	The consensus of the clinicians, pathologists, and radiologists	Physician diagnosis	CAD; HF	6
Kim et al. (27)	South Korea	SC	460	1,925 control subjects matched by age, gender and smoking habit	ATS/ERS/JRS/ALAT criteria (72)	Laboratory test	Acute IHD	7
Kizilirmak et al. (28)	Istanbul	SC	35	36 control subjects matched by age with post-COVID lung injury	The consensus of the clinicians, pathologists, and radiologists	Self-report	Unspecified CV diseases	6
López et al. (29)	Spain	MC	573	1,204 patients without IPF who underwent lung transplantation.	Validated ICD-10	The CMBD database	MI; HF; PH	5
Margaritopoulos et al. (30)	United Kingdom	SC	107	290 patients with ILD-non-IPF	The consensus of the clinicians, pathologists, and radiologists	Physician diagnosis	PH	6
Miyake et al. (31)	Japan	MC	104	56 acute bacterial pneumonia, and 4 common cold, matched by age, sex and time of visit	ATS/ERS criteria (68)	Chart review	CAD	7
Nathan et al. (32)	United States	SC	73	56 COPD lung transplant candidates matched by age	ATS/ERS criteria (71)	Physician diagnosis	CAD	7
Nolan et al. (33)	United Kingdom	SC	163	163 control subjects matched by age and gender with COPD	ATS/ERS/JRS/ALAT criteria (72)	Self-report	PH	6
Pedraza et al. (34)	Spain	MC	10,285	10,285 control subjects matched by age and gender	Diagnostic code (ICD-9)	The CMBD database	HF; VHD; PTE	5
Ponnuswamy et al. (35)	United Kingdom	SC	50	100 control subjects matched by age and gender	The consensus of the clinicians, pathologists, and radiologists	Chart review	AF; IHD	8
Sonaglioni et al. (36)	Italy	SC	105	102 control subjects matched by age and gender	ATS/ERS/JRS/ALAT criteria (2)	Chart review and laboratory test	CAS; CAD	7
Sonaglioni et al. (37)	Italy	SC	60	60 control subjects matched by age and gender	ATS/ERS/JRS/ALAT criteria (69)	Chart review and laboratory test	CAD; Arrhythmia	7
Sonaglioni et al. (38)	Italy	SC	50	30 control subjects matched by age and gender	ATS/ERS/JRS/ALAT criteria (69)	Chart review	CAD; VHD; Arrhythmia	7
Sun et al. (39)	China	SC	510	1,576 patients with CTD-ILD	Chart review	Chart review and laboratory test	MI; AF; PTE; VTE	6
Yalniz et al. (40)	Istanbul	SC	50	42 patients with ILD-non-IPF	ATS/ERS/JRS/ALAT criteria (72)	Self-report	CAD	5

MC, multicenter; SC: single center; IG, IPF group; CG, control group; ILD, interstitial lung disease; CTD: connective tissue disease; SSc: systemic sclerosis; COPD: chronic obstructive pulmonary disease; ATS: American Thoracic Society; ERS: European Respiratory Society; JRS: Japanese Respiratory Society; ALAT: Latin American Thoracic Association; PH: pulmonary hypertension; CAD: coronary artery disease; PTE: pulmonary thromboembolism; HF: heart failure; IHD: ischemic heart disease; AF: atrial fibrillation; MI: myocardial infarction; DVT: deep vein thrombosis; VHD: valvular heart disease; CAS: carotid artery stenosis; VTE: venous thromboembolism.

### 3.2 Meta-analysis

#### 3.2.1 Overall CV disease

**FIGURE 2:**
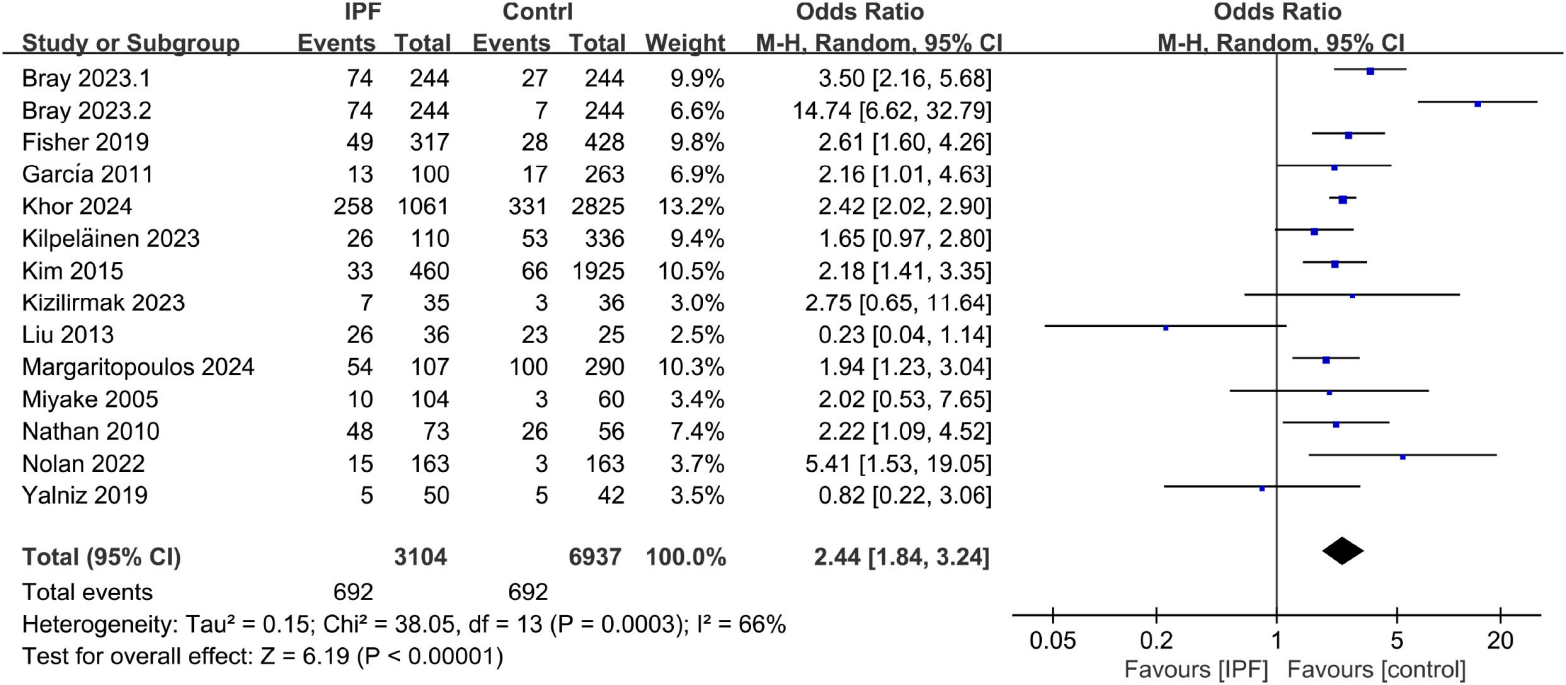
Prevalence of cardiovascular (CV) disease in idiopathic pulmonary fibrosis (IPF) (case-control/cross-sectional datasets).

**FIGURE 3:**
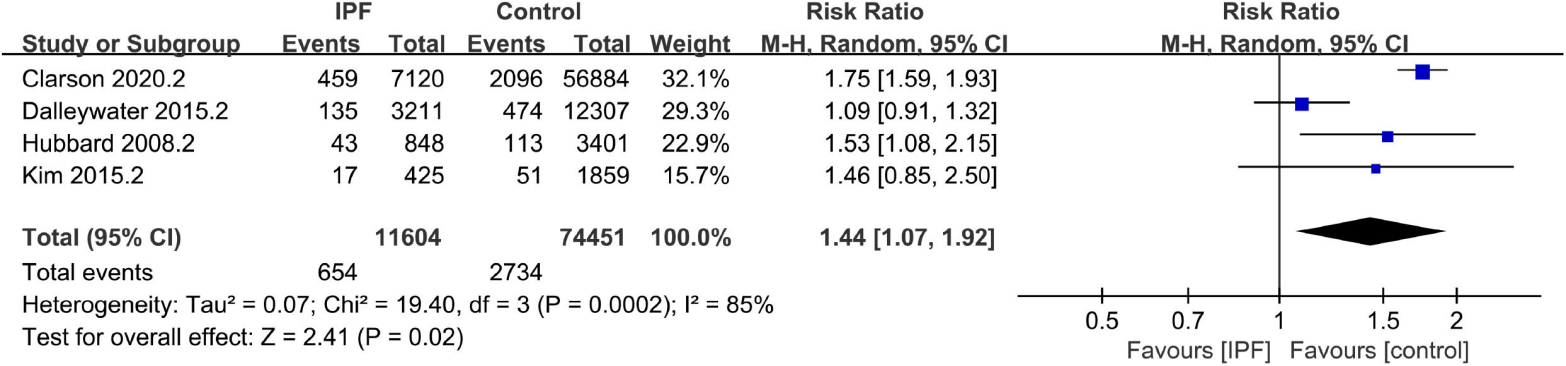
Prevalence of cardiovascular (CV) disease in idiopathic pulmonary fibrosis (IPF) (cohort datasets).

A total of 16 studies were eligible for the overall CV disease risk assessment, including those reporting either overall risk outcomes (22, 24, 25, 28) or only one specific outcome (13, 16, 19–21, 23, 27, 30–33, 40) (see Supplementary Material 6 for CV endpoints and pooled effect estimates across studies). Pooling the 14 case-control/cross-sectional datasets encompassed in these studies, the odds of CV disease diagnosis were significantly higher in patients with IPF than in those without (OR 2.44, 95% CI 1.84–3.24; *P* < 0.001, Figure 2). Meta-analysis of the four cohort datasets demonstrated that incident IPF significantly increased CV disease risk (RR 1.44, 95% CI 1.07–1.92; *P* = 0.02; Figure 3). Given the heterogeneity limitations (*I*^2^ = 66% and 85%, respectively), we conducted separate sensitivity analyses to ensure cautious interpretation of the results.

#### 3.2.2 Sensitivity analyses and meta-regression

Sensitivity analysis of the cohort datasets (Supplementary Material 7) identified one study (20) with self-reported CV disease outcomes as the primary source of heterogeneity. After removing this study, the fixed-effect meta-analysis yielded similar results (RR 1.72, 95% CI 1.57–1.89; *P* < 0.001) with complete resolution of heterogeneity (*I^2^* = 0%). However, sensitivity analysis failed to detect significant sources of bias in the case-control/cross-sectional datasets (Supplementary Material 8). We therefore performed meta-regression to investigate potential contributing factors (Supplementary Material 9). The 14 datasets were obtained from North America (*n* = 6), Asia (*n* = 5), and Europe (*n* = 3). Regression analysis by geographic region demonstrated non-significant results (*P* > 0.05), indicating that region was not a contributor to heterogeneity. Likewise, male proportion showed no association with heterogeneity (*P* > 0.05). The results demonstrated that control characteristics (*P* = 0.006), data source (*P* = 0.027), NOS score (*P* = 0.022), and certainty of CV disease diagnosis (*P* = 0.027) were significantly associated with CV disease risk in IPF patients, which may partially account for the observed heterogeneity.

#### 3.2.3 Subgroup analyses

**FIGURE 4:**
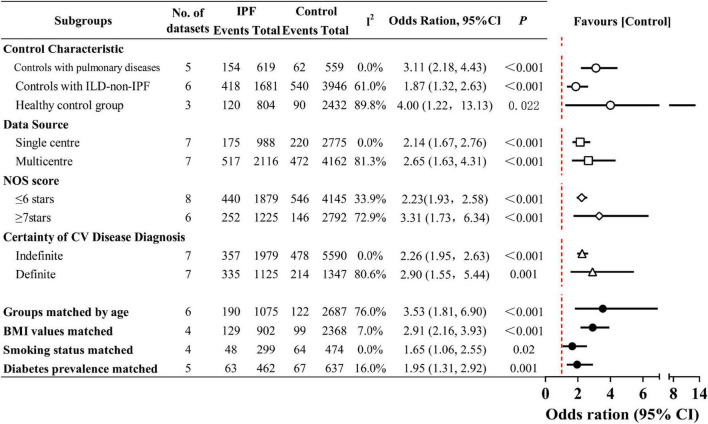
Prevalence of cardiovascular (CV) disease in idiopathic pulmonary fibrosis (IPF) by subgroup analyses. The dots indicate pooled relative risks. The horizontal lines indicate 95% confidence intervals for relative risks. The red dash vertical line marks the border for significance. Interstitial lung disease (ILD)-non-IPF: Other ILDs not caused by IPF.

As shown in Figure 4 (Supplementary Material 10), in the subgroup analyses, IPF was associated with a substantially higher risk of CV disease compared to non-ILD groups, including the matched healthy controls (OR 4.00; 95% CI 1.22–13.13; *P* = 0.022) and patients with other chronic pulmonary diseases (OR 3.11; 95% CI 2.18–4.43; *P* < 0.001). This observed variation may be due to the well-established association between ILD and CV disease (41). However, even after accounting for this, the association between IPF and CV disease was more pronounced than in ILD-non-IPF patients (OR 1.87; 95% CI 1.32–2.63; *P* < 0.001). Furthermore, multicenter design (OR 2.65, 95% CI 1.63–4.31; *P* < 0.001), definitive CV disease diagnosis (OR 2.90, 95% CI 1.55–5.44; *P* = 0.001), and higher NOS score (OR 3.31, 95% CI 1.73–6.34; *P* < 0.001) were each independently associated with higher CV disease risk. Studies with single center design (*I^2^* = 0%), indefinite CV disease diagnosis (*I^2^* = 0%), and lower NOS score (≤ 6; *I^2^* = 33.9%) all showed negligible to low heterogeneity, with stable pooled CV disease risk estimates [Single center (OR 2.14, 95% CI 1.67–2.76; *P* < 0.001), Indefinite diagnosis (2.26, 1.95–2.63; *P* < 0.001), NOS score ≤ 6 stars (2.23, 1.93–2.58; *P* < 0.001)].

Beyond regression-derived heterogeneity factors, all potential contributors to CV disease may introduce information bias, serving as potential sources of heterogeneity and confounding. Therefore, when assessing the adequacy of control subject selection, we considered age, BMI, smoking status, diabetes, hypertension, and dyslipidemia as covariates and performed subgroup analyses. When age was adjusted for in case and control groups, the heterogeneity existed as before (*I^2^* = 76.0%). Subgroup analyses using six age-balanced datasets showed higher pooled OR than the overall estimate (OR 3.53,95% CI 1.81–6.90; *P* < 0.001). The analysis revealed that the matched IPF cohorts had a mean age ranging from 60.1 to 73 years. This refined analysis more precisely characterizes the CV disease profile in elderly IPF patients, indicating a more severe CV burden in this aged IPF population. After matching the IPF and control groups for BMI (*I^2^* = 7%), smoking status (*I^2^* = 0%), and diabetes prevalence (*I^2^* = 16%), heterogeneity was effectively eliminated. Following comprehensive control of heterogeneity and adjustment for CV risk factors to eliminate outcome bias, the fixed-effects pooled analysis maintained a significant association without distorting the final estimates [BMI values matched (OR 2.91, 95% CI 2.16–3.93; *P* < 0.001), Smoking status matched (1.65, 1.06–2.55; *P* = 0.02), Diabetes prevalence matched (1.95, 1.31–2.92; *P* = 0.001)]. Still, two unresolved confounding factors (hypertension and dyslipidemia) were not adequately addressed in the included studies. Only one matched small-scale study (31) balanced the prevalence of both conditions and reported comparable CAD rates in IPF and pneumonia patients, leaving its conclusive significance unclear.

#### 3.2.4 Different categories of CV disease

**FIGURE 5:**
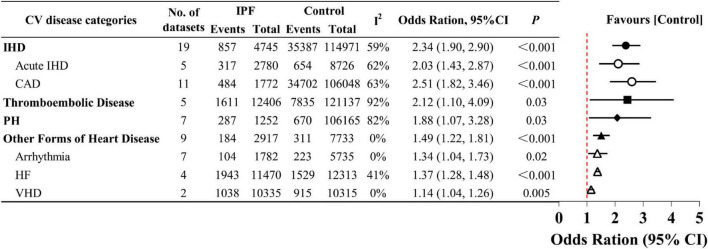
Prevalence of different categories of cardiovascular (CV) disease in idiopathic pulmonary fibrosis (IPF).

We assessed whether the OR varied by CV disease category (Figure 5 and Supplementary Material 11). Pooled analysis of 19 datasets (14–18, 21, 23, 26, 27, 29, 31, 32, 35–40) revealed significantly increased prevalence of IHD (OR 2.34, 95% CI 1.90–2.90; *P* < 0.001), with CAD (OR 2.51, 95% CI 1.82–3.46, *P* < 0.001) showing slightly higher risk than acute IHD (OR 2.03, 95% CI 1.43–2.87; *P* < 0.001). Notably, in the pooled analysis of five thromboembolic disease datasets from four studies (15, 23, 34, 39), IPF patients had a 2.12-fold higher risk than controls (OR 2.12, 95% CI 1.10–4.09; *P* = 0.003). Additionally, in the seven datasets presenting with PH identified (13–15, 18, 29, 30, 33), the risk of PH in patients with IPF almost doubled (OR 1.88, 95% CI 1.07–3.28; *P* = 0.03). Based on nine datasets (14, 17, 18, 23, 26, 29, 35, 37, 39), IPF patients showed a pooled 1.49-fold elevated risk of other forms of heart disease (95% CI 1.22–1.81; *P* < 0.001). Subgroup analyses further confirmed increased risks of arrhythmia (OR 1.34, 95% CI 1.04–1.73; *P* = 0.02) and HF (OR 1.37, 95% CI 1.28–1.48; *P* < 0.001). Moreover, compared with non-IPF, IPF demonstrated a significant albeit modest association with VHD (OR 1.14, 95% CI 1.04–1.26; *P* = 0.005).

In the fixed-effect meta-analysis of heart failure, the large-scale database study by Pedraza et al. (34) contributed 95.1% of the total weight, indicating severe weight imbalance that may dominate the pooled estimate. We suspect that the diagnosis of IPF in this study relied solely on ICD-9 code 516.3 without further validation (such as multidisciplinary discussion), potentially attenuating the true effect by including non-IPF pulmonary fibrosis cases. Our sensitivity analysis supported this hypothesis. After excluding the study in question, the pooled OR rose to 1.76 (95% CI 1.16–2.69; *P* = 0.008; data not shown). Additionally, with only two studies reporting VHD prevalence, the pooled risk estimate should be interpreted cautiously due to limited statistical power. Despite relatively low pooled risk estimates, the statistically significant associations (*P* < 0.05) consistently support a relationship between IPF and the other forms of heart disease, including its subtypes.

### 3.3 Publication bias

Using Stata, we evaluated publication bias for both overall and category-based CV diseases. The funnel plot distributions were generally symmetric (Supplementary Material 12), and Egger’s tests (Table 2) did not detect any obvious signs of systematic differences between small and large studies across the meta-analyses (all *P* > 0.05).

**TABLE 2:** Egger’s test for cardiovascular (CV) diseases.

CV disease categories	No. of datasets	Egger’s test (*p-*value)
Overall CV disease	14	0.862
IHD	19	0.364
Acute IHD	5	0.699
CAD	11	0.981
Thromboembolic disease	5	0.142
PH	7	0.36
Other forms of heart disease	9	0.794
Arrhythmia	7	0.799
HF	4	0.276
VHD	2	–

## 4 Discussion

### 4.1 Main findings and clinical inspiration

Idiopathic pulmonary fibrosis is a fatal disease that predominantly affects patients over 60 years of age (42). Patients in this age group are indeed at risk for multimorbidity, with CV disease being the second leading cause of death after IPF itself (43). For the crucial need to understand CV comorbidities associated with IPF, we conducted a systematic review and meta-analysis of existing observational studies. This work updates and expands the current understanding of the co-occurrence of CV disease and its major subtypes in IPF while quantifying risk estimates. To our knowledge, this represents the most comprehensive and potentially definitive systematic review to date.

Our research shows a significant correlation between IPF and CV disease, with an overall 2.44-fold increased risk compared to non-IPF populations. Given that the pooled risk estimates from cross-sectional/case-control datasets can only reflect the IPF-CV disease association at a specific time point, we additionally synthesized longitudinal evidence to verify their temporal relationship. The four cohort datasets prospectively recorded incident CV cases after accounting for baseline CV disease status, demonstrating that IPF onset or during its progression promotes subsequent CV development. However, our interpretation of this temporal association is constrained by limited data availability and may be influenced by random errors and insufficient statistical power, warranting cautious interpretation.

It has long been established that the heart and lung are linked both functionally and anatomically, underpinning the interconnection between IPF and CV disease. Notably, both diseases are quintessential age-related conditions (44). The pulmonary system is susceptible to cumulative damage with biological aging due to lifelong exposure to environmental challenges (45), while the heart is vulnerable owing to its high metabolic demand and infrequent cardiomyocyte turnover (46). Age-related biological processes such as senescence, inflammaging, autophagy, and mitochondrial dysfunction are interconnected (47), which diminish cardiopulmonary regenerative capacity and promote pathological fibrosis in both organs. Critically, chronic fibrosis represents a shared terminal pathway in both IPF and various CV diseases (48). Taken together, aging drives the co-progression of IPF and CV disease by establishing a pro-fibrotic microenvironment. Targeting these key age-related processes in future research may lead to better treatment approaches for cardiopulmonary comorbidities. Moreover, neural signaling also plays a non-negligible role in cardiopulmonary comorbidities. IPF may aggravate CV injury through autonomic nervous system dysregulation (49). Elucidating the fundamental mechanisms of neural control at the nerve-organ interface (heart and lung) could identify key regulatory circuits for therapeutic targeting.

Our analysis also encompassed multiple categories of CV disease. IHD represents a critical comorbidity in IPF patients, with CAD demonstrating the highest risk elevation (2.51-fold). A previous study by Raghu et al. (50) reported widely varying IHD prevalence rates (ranging from 3.2% for myocardial infarction to 68% for CAD) due to inconsistent definitions of IHD. To address this significant heterogeneity, our study provides the first dedicated risk estimate (OR = 2.03) for acute IHD. The mechanisms by which IPF is a risk factor for IHD remain unclear. One possibility is that impaired oxygen delivery may contribute to ischemia in the subendocardium, based on established cardiopulmonary pathophysiology. However, this hypothesis appears inconsistent with observations in COPD, where similar mechanisms do not result in elevated IHD risk (51). Thus, the IPF-IHD link is now thought to result from shared mechanisms, such as the shortening of telomeres (52).

The pooled risk of thromboembolic disease (OR = 2.12) followed closely behind, and emerged as a new risk category not previously reported in systematic reviews. Sode et al. (53) approached the relationship from a different perspective, demonstrating that the incidence of IPF was higher among individuals with a history of venous thromboembolism than those without. Epidemiological data have confirmed a bidirectional link between IPF and thrombosis. Mechanistic studies suggest that coagulation factors can directly promote fibrosis (54), while the fibrotic environment itself can activate thrombotic pathways (55), providing further biological evidence for this bidirectional relationship. This recognition spurred interest in targeting thrombotic pathways as a potential therapeutic strategy for IPF, yet prospective trials have generated concerning results (56). The current evidence recommends against anticoagulation unless indicated for other reasons. Particularly noteworthy is the emerging role of extracellular vesicles (EV) as key mediators in this complex pathophysiology (57). The therapeutic potential of EV-based approaches represents an exciting frontier in IPF treatment.

Furthermore, PH has long been recognized as a significant poor prognostic marker for IPF. Our study definitively demonstrates its increased risk in this patient population. The development of PH in IPF is usually a direct consequence of lung fibrotic destruction and hypoxic vasoconstriction (58), indicating there is (at least partially) a unidirectional causal relationship from IPF to PH. A large transcriptomic study (59) revealed a unique transcriptomic module positively linked to PH associated with IPF, showing shared gene expression patterns that implicate genetic susceptibility. Strikingly, this module directly influences metabolic changes in endothelial cells. Current evidence supports that (51) a detrimental cycle driven by persistent activation and cross-talk among endothelial (60), immune, inflammatory, and mesenchymal cells not only promotes fibrotic progression but also triggers and sustains vascular stiffening. Presently, there are no approved therapies for PH in IPF patients and the last two decades have seen a disappointing number of negative clinical trials using vasodilator therapies (61). Although approved for PH (62), sildenafil requires careful monitoring and is avoided in significant pulmonary parenchymal involvement.

Recently, Walters et al. (63) employed a single-arm meta-analysis to summarize comorbidities among participants of IPF-related RCTs. Interestingly, the reported CV comorbidities, including overall CV disease (23%, 95% CI 15%–33%), IHD (18%, 95% CI 13%–42%), and PH (4%, 95% CI 2%–6%), were consistently lower than our pooled estimates [25% (17%–33%), 21% (14%–30%), and 27% (15%–42%), respectively; data not shown]. We unexpectedly confirmed Walters et al.’s hypothesis that the reported comorbidity prevalence in IPF trial cohorts is lower than in observational studies, likely due to selection bias. The presence of comorbidities highlights two clinical concerns: (1) the necessity and risk of cardiovascular-antifibrotic drug combination (64), and (2) the risk of antifibrotic drug intolerance induced by concomitant medication burden (65). For example, nintedanib, a multi-target tyrosine kinase inhibitor (TKI), may increase bleeding risk when combined with anticoagulants (e.g., warfarin) (66). Concurrent use with calcium channel blockers (e.g., amiodarone) may elevate its plasma concentration via P-gp/CYP3A4 inhibition, potentially reducing tolerability (67). The RCT eligibility criteria excluding patients with CV diseases may compromise the applicability and translation of trial results to real-world patients with multimorbidity. Therefore, future RCTs in IPF should carefully weigh the potential risks and benefits of CV comorbidities.

Dalleywater et al. (20) reported increased CV risk in pre-diagnostic IPF patients, likely mediated by shared risk factors and common pathophysiological pathways. Integrating our findings with current understanding of the IPF-CV disease association, we recommend that IPF patients receive active management of CV risk factors alongside monitoring IPF progression. Specific measures may draw on established primary prevention interventions for CAD, such as smoking cessation, alcohol moderation, a healthy diet, regular exercise, weight control, and other evidence-based strategies. Regular imaging screenings should be performed to assess CV disease risk. Moreover, current evidence (19) suggests implementing risk-stratified CV prevention in IPF patients. For instance, more aggressive preventive measures are recommended for those aged < 50 years. As with IPF, most CV diseases, such as CAD, lack survival-prolonging therapies. Early detection and intervention may reduce disease burden and improve prognosis in IPF patients. For IPF patients diagnosed with CV disease, clinicians should not let the presence of severe pulmonary disease distract medical attention away from routine CV care. Standardized, proactive CV management yields significant clinical benefits to IPF patients at all disease stages.

### 4.2 Study limitations

In our meta-analysis, we screened literature in strict accordance with inclusion and exclusion criteria, and implemented a high-quality study design. The overall consistency of evidence supports a real association between IPF and CV disease. Nevertheless, there remain several limitations in our study. The inclusion of observational studies with varying diagnostic criteria, clinical settings, and study designs resulted in statistically significant heterogeneity. To overcome this limitation, we maximized subgroup analyses based on available research evidence by incorporating both meta-regression findings and CV disease risk factors wherever feasible. The consistent findings after controlling for heterogeneity and bias further substantiated the reliability of our results.

However, retrospective datasets are particularly vulnerable to multiple confounding factors, including recall bias. Unadjusted covariates such as hypertension and dyslipidemia may obscure the association between IPF and CV disease, especially considering the conditions’ shared risk factors. Sensitivity analyses and publication bias tests suggest that sampling biases are unlikely to be responsible for the observed associations. Nevertheless, selection bias remains possible since the included studies recruited participants based on a combination of different factors. Thus, caution is warranted when extrapolating these results to the overall IPF population. Furthermore, 12 of the 28 included studies utilized medical registration datasets. Although some implemented clinical validation through prescription records, medical history, or equivalent verification methods to reduce bias, these datasets carry inherent risks of inaccurate and/or incomplete coding. This shortcoming could lead to an underestimation of the true disease prevalence.

In addition, most studies of the IPF-CV disease link are retrospective; thus, our conclusions rely mainly on case–control or cross-sectional data that can indicate an association but cannot establish causation. Any attempt at causal inference is currently limited to four prospective cohort datasets. Though these studies provide preliminary insights, the limited number warrants caution in interpreting the observed temporal relationship. Therefore, the robustness of unidirectional causal inference between IPF and CV disease remains insufficient. We strongly encourage additional high-quality prospective cohort studies to evaluate the bidirectional causal relationship between IPF and CV disease. Such studies would clarify both the directionality and temporal dynamics of these associations.

## 5 Conclusion

Taken together, this meta-analysis of observational studies found that IPF patients have an elevated risk of CV events. This risk derives from increased risk of IHD, thrombotic disease, PH and other forms of heart disease. Longitudinal evidence suggests that IPF may be a risk factor for CV disease, but the causal link requires careful evaluation. These findings highlight the need to raise awareness about the coexistence of IPF and CV disease and define the best clinical and cost-effectiveness frameworks for screening and developing intervention strategies for CV risks in patients with IPF.

## Data Availability

The original contributions presented in this study are included in this article/Supplementary material, further inquiries can be directed to the corresponding authors.

## References

[1] Vancheri C Failla M Crimi N Raghu G. Idiopathic pulmonary fibrosis: a disease with similarities and links to cancer biology. *Eur Respir J.* (2010) 3:496–504. 10.1183/09031936.00077309 20190329

[2] Raghu G Remy-Jardin M Richeldi L Thomson C Inoue Y Johkoh T 1 idiopathic pulmonary fibrosis (an update) and progressive pulmonary fibrosis in adults: an official ATS/ERS/JRS/ALAT clinical practice guideline. *Am J Respir Crit Care Med.* (2022) 9:e18–47. 10.1164/rccm.202202-0399ST 35486072 PMC9851481

[3] Nalysnyk L Cid-Ruzafa J Rotella P Esser D. Incidence and prevalence of idiopathic pulmonary fibrosis: a review of the literature. *Eur Respir Rev.* (2012) 126:355–61. 10.1183/09059180.00002512 23204124 PMC9487229

[4] Torrisi S Ley B Kreuter M Wijsenbeek M Vittinghoff E Collard H The added value of comorbidities in predicting survival in idiopathic pulmonary fibrosis: a multicentre observational study. *Eur Respir J.* (2019) 3:1801587. 10.1183/13993003.01587-2018 30578385

[5] Caminati A Lonati C Cassandro R Elia D Pelosi G Torre O Comorbidities in idiopathic pulmonary fibrosis: an underestimated issue. *Eur Respir Rev.* (2019) 153:190044. 10.1183/16000617.0044-2019 31578211 PMC9488913

[6] Oldham J Collard H. Comorbid conditions in idiopathic pulmonary fibrosis: recognition and management. *Front Med.* (2017) 4:123. 10.3389/fmed.2017.00123 28824912 PMC5539138

[7] Hayes D Black S Tobias J Kirkby S Mansour H Whitson B. Influence of pulmonary hypertension on patients with idiopathic pulmonary fibrosis awaiting lung transplantation. *Ann Thorac Surg.* (2016) 1:246–52. 10.1016/jathoracsur.2015.06.024 26319484

[8] Kärkkäinen M Kettunen H Nurmi H Selander T Purokivi M Kaarteenaho R. Effect of smoking and comorbidities on survival in idiopathic pulmonary fibrosis. *Respir Res.* (2017) 1:160. 10.1186/s12931-017-0642-6 28830514 PMC5567897

[9] Kreuter M Bonella F Maher T Costabel U Spagnolo P Weycker D Effect of statins on disease-related outcomes in patients with idiopathic pulmonary fibrosis. *Thorax.* (2017) 2:148–53. 10.1136/thoraxjnl-2016-208819 27708114 PMC5284334

[10] Vedel-Krogh S Nielsen S Nordestgaard B. Statin use is associated with reduced mortality in patients with interstitial lung disease. *PLoS One.* (2015) 10:e0140571. 10.1371/journal.pone.0140571 26473476 PMC4608706

[11] Kreuter M Costabel U Richeldi L Cottin V Wijsenbeek M Bonella F Statin therapy and outcomes in trials of nintedanib in idiopathic pulmonary fibrosis. *Respiration.* (2018) 5:317–26. 10.1159/000486286 29414827

[12] Page M McKenzie J Bossuyt P Boutron I Hoffmann T Mulrow C The PRISMA 2020 statement: an updated guideline for reporting systematic reviews. *BMJ.* (2021) 372:71. 10.1136/bmj.n71 33782057 PMC8005924

[13] Fang L Ran W Aipin B Xuewei F. Comparison of the clinical features of microscopic polyangiitis with pulmonary fibrosis as the first manifestation and idiopathic pulmonary fibrosis. *Chin J Postgrad Med.* (2013) 1:42–4. 10.3760/cma.j.issn.1673-4904.2013.01.016

[14] Ba C Wang H Jiang C Shi X Jin J Fang Q. Clinical manifestations and prognostic factors analysis of patients hospitalized with acute exacerbation of idiopathic pulmonary fibrosis and other interstitial lung diseases. *BMJ Open Respir Res.* (2024) 1:e001997. 10.1136/bmjresp-2023-001997 38413119 PMC10900369

[15] Bade B Shojaee A Gulati M. Hospital-associated outcomes for IPF patients with lung cancer. *Am J Respir Crit Care Med.* (2019) 199:8312. 10.1164/ajrccm-conference.2019.199.1

[16] Bray K Bodduluri S Kim Y Sthanam V Nath H Bhatt SP. Idiopathic pulmonary fibrosis is more strongly associated with coronary artery disease than chronic obstructive pulmonary disease. *Respiratory Med.* (2023) 211:107195. 10.1016/j.rmed.2023.107195 36889520 PMC10122707

[17] Canıvar C Bingöl Z Kılıçaslan Z Çağatay T Okumuş N. Investigation of parameters related to prognosis in diffuse parenchymal lung diseases prognosis in interstitial lung diseases. *Tuberk Toraks.* (2017) 3:210–9. 10.5578/tt.57501 29135399

[18] Chan R Horrigan M Goh N Khor Y. Clinical assessment for pulmonary hypertension in interstitial lung disease. *Intern Med J.* (2023) 8:1415–22. 10.1111/imj.15887 35848362

[19] Clarson L Bajpai R Whittle R Belcher J Abdul Sultan A Kwok C Interstitial lung disease is a risk factor for ischaemic heart disease and myocardial infarction. *Heart.* (2020) 12:916–22. 10.1136/heartjnl-2019-315511 32114515 PMC7282497

[20] Dalleywater W Powell H Hubbard R Navaratnam V. Risk factors for cardiovascular disease in people with idiopathic pulmonary fibrosis: a population-based study. *Chest.* (2015) 1:150–6. 10.1378/chest.14-0041 25121965

[21] Fisher J Kolb M Algamdi M Morisset J Johannson K Shapera S Baseline characteristics and comorbidities in the Canadian REgistry for pulmonary fibrosis. *BMC Pulm Med.* (2019) 1:223. 10.1186/s12890-019-0986-4 31771541 PMC6880596

[22] García-Sancho C Buendía-Roldán I Navarro C Pérez-Padilla R Vargas MH Fernández-plata MaR. *Respiratory Med.* (2011) 12:1902–7. 10.1016/j.rmed.2011.08.022 21917441

[23] Hubbard R Smith C Le Jeune I Gribbin J Fogarty A. The association between idiopathic pulmonary fibrosis and vascular disease: a population-based study. *Am J Respir Crit Care Med.* (2008) 12:1257–61. 10.1164/rccm.200805-725OC 18755924

[24] Khor Y Johannson K Marcoux V Fisher J Assayag D Manganas H Epidemiology and prognostic significance of cough in fibrotic interstitial lung disease. *Am J Respir Crit Care Med.* (2024) 8:1035–44. 10.1164/rccm.202311-2101OC 38536110

[25] Kilpeläinen M Hirvonen T Perkonoja K Hirsjärvi S. Clinical characteristics and disease course of fibrosing interstitial lung disease patients in a real-world setting. *Medicina.* (2023) 2:281. 10.3390/medicina59020281 36837481 PMC9961403

[26] Kato S Kitamura H Hayakawa K Fukui K Tabata E Otoshi R Coronary artery disease and heart failure in patients with idiopathic pulmonary fibrosis. *Heart Vessels.* (2021) 8:1151–8. 10.1007/s00380-021-01787-1 33486554

[27] Kim W Mok Y Kim G Baek S Yun Y Jee S Association between idiopathic pulmonary fibrosis and coronary artery disease: a case-control study and cohort analysis. *Sarcoidosis Vasc Diffuse Lung Dis.* (2015) 4:289–96. 25591140

[28] Kızılırmak D Sarı S Can F Havlucu Y. Radiological findings based comparison of functional status in patients who have post-covid lung injury or idiopathic pulmonary fibrosis. *BMC Pulm Med.* (2023) 1:234. 10.1186/s12890-023-02527-z 37391786 PMC10314393

[29] López-Muñiz Ballesteros B Lopez-de-Andres A Jimenez-Garcia R Zamorano-Leon J Carabantes-Alarcon D Cuadrado-Corrales N Trends and outcomes in lung transplantation in patients with and without idiopathic pulmonary fibrosis in Spain during the Period 2016-2020. *Healthcare.* (2023) 11:1534. 10.3390/healthcare11111534 37297674 PMC10252271

[30] Margaritopoulos G Proklou A Trachalaki A Badenes Bonet D Kokosi M Kouranos V Overnight desaturation in interstitial lung diseases: links to pulmonary vasculopathy and mortality. *ERJ Open Res.* (2024) 1:00740–2023. 10.1183/23120541.00740-2023 38348245 PMC10860199

[31] Miyake Y Sasaki S Yokoyama T Chida K Azuma A Suda T Case-control study of medical history and idiopathic pulmonary fibrosis in Japan. *Respirology.* (2005) 4:504–9. 10.1111/j.1440-1843.2005.00742.x 16135175

[32] Nathan S Basavaraj A Reichner C Shlobin O Ahmad S Kiernan J Prevalence and impact of coronary artery disease in idiopathic pulmonary fibrosis. *Respir Med.* (2010) 7:1035–41. 10.1016/j.rmed.2010.02.008 20199856

[33] Nolan C Polgar O Schofield S Patel S Barker R Walsh J Pulmonary rehabilitation in idiopathic pulmonary fibrosis and COPD: a propensity-matched real-world study. *Chest.* (2022) 3:728–37. 10.1016/j.chest.2021.10.021 34699771 PMC8941605

[34] Pedraza-Serrano F Jiménez-García R López-de-Andrés A Hernández-Barrera V Esteban-Hernández J Sánchez-Muñoz G 67 Comorbidities and risk of mortality among hospitalized patients with idiopathic pulmonary fibrosis in Spain from 2002 to 2014. *Respir Med.* (2018) 138:137–43. 10.1016/j.rmed.2018.04.005 29724386

[35] Ponnuswamy A Manikandan R Sabetpour A Keeping I Finnerty J. Association between ischaemic heart disease and interstitial lung disease: a case-control study. *Respir Med.* (2009) 4:503–7. 10.1016/j.rmed.2009.01.004 19196502

[36] Sonaglioni A Caminati A Behring G Nicolosi G Rispoli G Zompatori M Prognostic role and determinants of ascending aorta dilatation in non-advanced idiopathic pulmonary fibrosis: a preliminary observation from a tertiary university center. *J Clin Med.* (2025) 4:1300. 10.3390/jcm14041300 40004830 PMC11856476

[37] Sonaglioni A Caminati A Lipsi R Lombardo M Harari S. Association between C-reactive protein and carotid plaque in mild-to-moderate idiopathic pulmonary fibrosis. *Intern Emerg Med.* (2021) 6:1529–39. 10.1007/s11739-020-02607-6 33411265

[38] Sonaglioni A Caminati A Lipsi R Nicolosi G Lombardo M Anzà C Early left atrial dysfunction in idiopathic pulmonary fibrosis patients without chronic right heart failure. *Int J Cardiovasc Imaging.* (2020) 9:1711–23. 10.1007/s10554-020-01887-5 32448985

[39] Sun H Liu M Yang X Xi L Xu W Deng M Incidence and risk factors of venous thrombotic events in patients with interstitial lung disease during hospitalization. *Thromb J.* (2023) 1:17. 10.1186/s12959-023-00458-7 36765371 PMC9912624

[40] Yalnız E Polat G Demirci F Deniz S Karadeniz G Aydınlı E Are idiopathic pulmonary fibrosis patients more anxious and depressive than patients with other interstitial lung disease? *Sarcoidosis Vasc Diffuse Lung Dis.* (2019) 4:294–301. 10.36141/svdld.v36i4.8418 32476965 PMC7247093

[41] Hu Z Wang H Huang J Yang G Luo W Zhong J Cardiovascular disease in connective tissue disease-associated interstitial lung disease: a systematic review and meta-analysis of observational studies. *Autoimmun Rev.* (2024) 10:103614. 10.1016/j.autrev.2024.103614 39222675

[42] Richeldi L Collard H Jones M. Idiopathic pulmonary fibrosis. *Lancet.* (2017) 10082:1941–52. 10.1016/S0140-6736(17)30866-8 28365056

[43] Rajala K Lehto J Saarinen M Sutinen E Saarto T Myllärniemi M. End-of-life care of patients with idiopathic pulmonary fibrosis. *BMC Palliat Care.* (2016) 1:85. 10.1186/s12904-016-0158-8 27729035 PMC5059981

[44] Newman A Arnold A Naydeck B Fried L Burke G Enright P Successful aging: effect of subclinical cardiovascular disease. *Arch Intern Med.* (2003) 19:2315–22. 10.1001/archinte.163.19.2315 14581251

[45] Chilosi M Carloni A Rossi A Poletti V. Premature lung aging and cellular senescence in the pathogenesis of idiopathic pulmonary fibrosis and COPD/emphysema. *Transl Res.* (2013) 3:156–73. 10.1016/j.trsl.2013.06.004 23831269

[46] Linton P Gurney M Sengstock D Mentzer R Gottlieb R. This old heart: cardiac aging and autophagy. *J Mol Cell Cardiol.* (2015) 83:44–54. 10.1016/j.yjmcc.2014.12.017 25543002 PMC4459942

[47] Murtha L Morten M Schuliga M Mabotuwana N Hardy S Waters D The role of pathological aging in cardiac and pulmonary fibrosis. *Aging Dis.* (2019) 2:419–28. 10.14336/AD.2018.0601 31011486 PMC6457057

[48] Murtha L Schuliga M Mabotuwana N Hardy S Waters D Burgess J The processes and mechanisms of cardiac and pulmonary fibrosis. *Front Physiol.* (2017) 8:777. 10.3389/fphys.2017.00777 29075197 PMC5643461

[49] Mehra R Tjurmina O Ajijola O Arora R Bolser D Chapleau M Research opportunities in autonomic neural mechanisms of cardiopulmonary regulation: a report from the national heart, lung, and blood institute and the national institutes of health office of the director workshop. *JACC Basic Transl Sci.* (2022) 3:265–93. 10.1016/j.jacbts.2021.11.003 35411324 PMC8993767

[50] Raghu G Amatto V Behr J Stowasser S. Comorbidities in idiopathic pulmonary fibrosis patients: a systematic literature review. *Eur Respiratory J.* (2015) 4:1113–30. 10.1183/13993003.02316-2014 26424523

[51] Selman M Buendia-Roldan I Pardo A. Decoding the complexity: mechanistic insights into comorbidities in idiopathic pulmonary fibrosis. *Eur Respiratory J.* (2025) 65:2402418. 10.1183/13993003.02418-2024 40180336 PMC12095908

[52] Chen B Yan Y Wang H Xu J. Association between genetically determined telomere length and health-related outcomes: a systematic review and meta-analysis of Mendelian randomization studies. *Aging Cell.* (2023) 7:e13874. 10.1111/acel.13874 37232505 PMC10352568

[53] Sode B Dahl M Nielsen S Nordestgaard B. Venous thromboembolism and risk of idiopathic interstitial pneumonia: a nationwide study. *Am J Respir Crit Care Med.* (2010) 10:1085–92. 10.1164/rccm.200912-1951OC 20167844

[54] Isermann B. Homeostatic effects of coagulation protease-dependent signaling and protease activated receptors. *J Thromb Haemost.* (2017) 7:1273–84. 10.1111/jth.13721 28671351

[55] Isshiki T Sakamoto S Homma S. Therapeutic role of recombinant human soluble thrombomodulin for acute exacerbation of idiopathic pulmonary fibrosis. *Medicina.* (2019) 5:172. 10.3390/medicina55050172 31137593 PMC6571552

[56] Tomassetti S Ruy J Gurioli C Ravaglia C Buccioli M Tantalocco P The effect of anticoagulant therapy for idiopathic pulmonary fibrosis in real life practice. *Sarcoidosis Vasc Diffuse Lung Dis.* (2013) 2:121–7. 24071883

[57] Jansen F Nickenig G Werner N. Extracellular vesicles in cardiovascular disease: potential applications in diagnosis, prognosis, and epidemiology. *Circ Res.* (2017) 10:1649–57. 10.1161/CIRCRESAHA.117.310752 28495995

[58] Harder E Abtin F Nardelli P Brownstein A Channick R Washko G Pulmonary hypertension in idiopathic interstitial pneumonia is associated with small vessel pruning. *Am J Respir Crit Care Med.* (2024) 9:1170–3. 10.1164/rccm.202312-2343LE 38502314 PMC11092950

[59] Brownstein A Mura M Ruffenach G Channick R Saggar R Kim A Dissecting the lung transcriptome of pulmonary fibrosis-associated pulmonary hypertension. *Am J Physiol Lung Cell Mol Physiol.* (2024) 4:L520–34. 10.1152/ajplung.00166.2024 39137526 PMC11482468

[60] Fließer E Jandl K Lins T Birnhuber A Valzano F Kolb D Lung fibrosis is linked to increased endothelial cell activation and dysfunctional vascular barrier integrity. *Am J Respir Cell Mol Biol.* (2024) 3:318–31. 10.1165/rcmb.2024-0046OC 38843440

[61] Harari S Elia D Humbert M. Pulmonary hypertension in parenchymal lung diseases: any future for new therapies? *Chest.* (2018) 1:217–23. 10.1016/j.chest.2017.06.008 28629920

[62] Hoeper M Welte T. Sildenafil citrate therapy for pulmonary arterial hypertension. *N Engl J Med.* (2006) 10:1091–3. 10.1056/NEJMc053442 16525151

[63] Walters T Leong M Montesi S Ryerson C Khor Y. Comorbidities in the idiopathic pulmonary fibrosis and progressive pulmonary fibrosis trial population: a systematic review and meta-analysis. *Eur Respir Rev.* (2025) 175:240238. 10.1183/16000617.0238-2024 40107663 PMC11920886

[64] Zhang W Wang X Xue C Ji X Pan L Weng W The effect of cardiovascular medications on disease-related outcomes in idiopathic pulmonary fibrosis: a systematic review and meta-analysis. *Front Pharmacol.* (2021) 12:771804. 10.3389/fphar.2021.771804 34858190 PMC8632524

[65] Khor Y Goh N Wong A Johannson K Marcoux V Fisher J Impact of concomitant medication burden on tolerability of disease-targeted therapy and survival in interstitial lung disease. *Ann Am Thorac Soc.* (2022) 6:962–70. 10.1513/AnnalsATS.202108-980OC 35007498

[66] Grześk G Woźniak W Błażejewski J Górny B Wołowiec Ł Rogowicz D The interactions of nintedanib and oral anticoagulants-molecular mechanisms and clinical implications. *Int J Mol Sci.* (2020) 22:282. 10.3390/ijms22010282 33396592 PMC7795697

[67] Shah R Morganroth J. Update on cardiovascular safety of tyrosine kinase inhibitors: with a special focus on QT interval, left ventricular dysfunction and overall risk/benefit. *Drug Saf.* (2015) 38:693–710. 10.1007/s40264-015-0300-1 26008987

[68] Demedts M Costabel U. ATS/ERS international multidisciplinary consensus classification of the idiopathic interstitial pneumonias. *Eur Respir J.* (2002) 5:794–6. 10.1183/(09031936):02.00492002. 12030715

[69] Raghu G Remy-Jardin M Myers J Richeldi L Ryerson C Lederer D Diagnosis of idiopathic pulmonary fibrosis. An official ATS/ERS/JRS/ALAT clinical practice guideline. *Am J Respir Crit Care Med.* (2018) 5:e44–68. 10.1164/rccm.201807-1255ST 30168753

[70] Thomson C Duggal A Bice T Lederer D Wilson K Raghu G. 2018 clinical practice guideline summary for clinicians: diagnosis of idiopathic pulmonary fibrosis. *Ann Am Thorac Soc.* (2019) 3:285–90. 10.1513/AnnalsATS.201809-604CME 30543449

[71] American Journal of Respiratory and Critical Care Medicine. American Thoracic Society. Idiopathic pulmonary fibrosis: diagnosis and treatment. International consensus statement. American thoracic society (ATS), and the European respiratory society (ERS). *Am J Respir Crit Care Med.* (2000) 2:646–64. 10.1164/ajrccm.161.2.ats3-00 10673212

[72] Raghu G Collard H Egan J Martinez F Behr J Brown K An official ATS/ERS/JRS/ALAT statement: idiopathic pulmonary fibrosis: evidence-based guidelines for diagnosis and management. *Am J Respir Crit Care Med.* (2011) 6:788–824. 10.1164/rccm.2009-040GL 21471066 PMC5450933

