# Supplementary material for: Cardiovascular disease in idiopathic pulmonary fibrosis: a systematic review and meta-analysis of observational studies
Source: Front Med (Lausanne). 2025 Sep 22;12:1653435. doi: 10.3389/fmed.2025.1653435 (PMC12497613; doi:10.3389/fmed.2025.1653435)

Supplementary Material

Cardiovascular Disease in Idiopathic Pulmonary Fibrosis: A Systematic Review and Meta-Analysis of Observational Studies

Yang Li[1†], Weili Tan[1†], Yangyini Zhang[1], Fang Cao[2], Zhisong Wu[3], Yang Jiao[4*], Jie Niu[2*]

*** Correspondence:**
Yang Jiao: yangjiao2013@sina.cn
Jie Niu* niujie_work@126.com

† Equal contributions:
Yang Li[1†] and Weili Tan[1†] two authors contributed equally to this work and share first authorship.

Supplementary Material S1. PRISMA 2020 Checklist

Section and Topic	Item #	Checklist item	Location where item is reported
**TITLE**			
Title	1	Identify the report as a systematic review.	1
**ABSTRACT**			
Abstract	2	See the PRISMA 2020 for Abstracts checklist.	none
**INTRODUCTION**			
Rationale	3	Describe the rationale for the review in the context of existing knowledge.	2
Objectives	4	Provide an explicit statement of the objective(s) or question(s) the review addresses.	2
**METHODS**			
Eligibility criteria	5	Specify the inclusion and exclusion criteria for the review and how studies were grouped for the syntheses.	3
Information sources	6	Specify all databases, registers, websites, organisations, reference lists and other sources searched or consulted to identify studies. Specify the date when each source was last searched or consulted.	2-3
Search strategy	7	Present the full search strategies for all databases, registers and websites, including any filters and limits used.	2-3
Selection process	8	Specify the methods used to decide whether a study met the inclusion criteria of the review, including how many reviewers screened each record and each report retrieved, whether they worked independently, and if applicable, details of automation tools used in the process.	3
Data collection process	9	Specify the methods used to collect data from reports, including how many reviewers collected data from each report, whether they worked independently, any processes for obtaining or confirming data from study investigators, and if applicable, details of automation tools used in the process.	3
Data items	10a	List and define all outcomes for which data were sought. Specify whether all results that were compatible with each outcome domain in each study were sought (e.g. for all measures, time points, analyses), and if not, the methods used to decide which results to collect.	3
	10b	List and define all other variables for which data were sought (e.g. participant and intervention characteristics, funding sources). Describe any assumptions made about any missing or unclear information.	3
Study risk of bias assessment	11	Specify the methods used to assess risk of bias in the included studies, including details of the tool(s) used, how many reviewers assessed each study and whether they worked independently, and if applicable, details of automation tools used in the process.	3
Effect measures	12	Specify for each outcome the effect measure(s) (e.g. risk ratio, mean difference) used in the synthesis or presentation of results.	3
Synthesis methods	13a	Describe the processes used to decide which studies were eligible for each synthesis (e.g. tabulating the study intervention characteristics and comparing against the planned groups for each synthesis (item #5)).	3
	13b	Describe any methods required to prepare the data for presentation or synthesis, such as handling of missing summary statistics, or data conversions.	3
	13c	Describe any methods used to tabulate or visually display results of individual studies and syntheses.	3
	13d	Describe any methods used to synthesize results and provide a rationale for the choice(s). If meta-analysis was performed, describe the model(s), method(s) to identify the presence and extent of statistical heterogeneity, and software package(s) used.	3
	13e	Describe any methods used to explore possible causes of heterogeneity among study results (e.g. subgroup analysis, meta-regression).	3
	13f	Describe any sensitivity analyses conducted to assess robustness of the synthesized results.	3

Section and Topic	Item #	Checklist item	Location where item is reported
Reporting bias assessment	14	Describe any methods used to assess risk of bias due to missing results in a synthesis (arising from reporting biases).	3
Certainty assessment	15	Describe any methods used to assess certainty (or confidence) in the body of evidence for an outcome.	3
**RESULTS**			
Study selection	16a	Describe the results of the search and selection process, from the number of records identified in the search to the number of studies included in the review, ideally using a flow diagram.	4 and figure 1
	16b	Cite studies that might appear to meet the inclusion criteria, but which were excluded, and explain why they were excluded.	4
Study characteristics	17	Cite each included study and present its characteristics.	4 and table1
Risk of bias in studies	18	Present assessments of risk of bias for each included study.	4
Results of individual studies	19	For all outcomes, present, for each study: (a) summary statistics for each group (where appropriate) and (b) an effect estimate and its precision (e.g. confidence/credible interval), ideally using structured tables or plots.	4-5
Results of syntheses	20a	For each synthesis, briefly summarise the characteristics and risk of bias among contributing studies.	4
	20b	Present results of all statistical syntheses conducted. If meta-analysis was done, present for each the summary estimate and its precision (e.g. confidence/credible interval) and measures of statistical heterogeneity. If comparing groups, describe the direction of the effect.	4-5
	20c	Present results of all investigations of possible causes of heterogeneity among study results.	4-5
	20d	Present results of all sensitivity analyses conducted to assess the robustness of the synthesized results.	4
Reporting biases	21	Present assessments of risk of bias due to missing results (arising from reporting biases) for each synthesis assessed.	5
Certainty of evidence	22	Present assessments of certainty (or confidence) in the body of evidence for each outcome assessed.	5
**DISCUSSION**			
Discussion	23a	Provide a general interpretation of the results in the context of other evidence.	5-7
	23b	Discuss any limitations of the evidence included in the review.	7
	23c	Discuss any limitations of the review processes used.	7
	23d	Discuss implications of the results for practice, policy, and future research.	7
**OTHER INFORMATION**			
Registration and protocol	24a	Provide registration information for the review, including register name and registration number, or state that the review was not registered.	2
	24b	Indicate where the review protocol can be accessed, or state that a protocol was not prepared.	2
	24c	Describe and explain any amendments to information provided at registration or in the protocol.	none
Support	25	Describe sources of financial or non-financial support for the review, and the role of the funders or sponsors in the review.	10
Competing interests	26	Declare any competing interests of review authors.	10
Availability of data, code and other materials	27	Report which of the following are publicly available and where they can be found: template data collection forms; data extracted from included studies; data used for all analyses; analytic code; any other materials used in the review.	10

Supplementary Material S2. Detailed description of the search strategy

PubMed	
#1	‘idiopathic pulmonary fibrosis’ [MeSH Terms]
#2	‘idiopathic pulmonary fibroses’ OR ‘pulmonary fibroses, idiopathic’ OR ‘pulmonary fibrosis, idiopathic’ OR ‘usual interstitial pneumonia’ OR ‘pulmonary fibrosis’ OR ‘fibrosis, pulmonary’ OR ‘IPF’
#3	‘cardiovascular diseases’ [MeSH Terms]
#4	‘cardiovascular disease’ OR ‘disease, cardiovascular’ OR ‘cardiac event’
#5	‘cerebrovascular disorders’ OR ‘hypertension’ OR ‘acute coronary syndrome’ OR ‘myocardial ischemia’ OR ‘ischemic heart disease’ OR ‘IHD’ OR ‘acute coronary syndrome’ OR ‘angina pectoris’ OR ‘myocardial infarction’ OR ‘MI’ OR ‘coronary thrombosis’ OR ‘coronary artery disease’ OR ‘CAD’ OR ‘pulmonary heart disease’ OR ‘pulmonary hypertension’ OR ‘PH’ OR ‘thromboembolic disease’ OR ‘pulmonary thromboembolism’ OR ‘deep vein thrombosis’ OR ‘venous thromboembolism’ OR ‘heart valve diseases’ OR ‘heart failure’ OR ‘atrial fibrillation’ OR ‘arrhythmia’ OR ‘myocarditis’ OR ‘pericardial effusion’ OR ‘endocarditis’ OR ‘pericarditis’
#6	‘comorbidity’ [MeSH Terms]
#7	‘co-morbidity’ OR ‘co-morbidities’
#8	‘case-control Studies’ OR ‘cohort studies’ OR ‘cross-sectional studies’ OR ‘follow-up’ OR ‘prospective’ OR ‘retrospective’
#9	‘search*’ OR ‘meta-analysis’ OR ‘meta analysis as topic’ OR ‘review’ OR ‘diagnosis’ OR ‘interview*’ OR ‘interviews as topic’ OR ‘experience*’
#10	#1 OR #2
#11	#3 OR #4 OR #5 OR #6 OR #7
#12	#8 NOT #9
#13	#10 AND #11 AND #12

Supplementary Material S3. The definition of CV disease

All datasets using any of the following methods of defining CV disease were included:
1) laboratory test, physician diagnosis, or medical chart review
2) administrative data or similar sources, with clinical validation through prescription records, medical history, or equivalent verification methods.
3) unvalidated administrative data or similar sources.
4) data sourced from unverified self-reports
Definitions 1-2 were accepted as definite CV disease, whereas 3-4 were indefinite.

Supplementary Material S4. Cardiovascular comorbidity categories

1) Unspecified CV disease is a broad category that includes any CV diseases collected and reported;
2) Ischemic Heart Disease (IHD): ICD-11 BA40-6Z, 81-8Z; ICD-10 I20-I25;
2A) Coronary Artery Disease (CAD): ICD-11 BA52, 81-8Z; ICD-10 I25.1, I25.4, I25.9;
2B) Acute IHD (Acute Coronary Syndrome): ICD-11 BA40-4Z; ICD-10 I20-22, 24;
3) Thromboembolic Disease: ICD-11 BB00, BD71-72; ICD-10 I26, I81-82;
4) Pulmonary Hypertension (PH): ICD-11 BB01.2; ICD-10 I27.2;
5) Other Forms of Heart Disease: BB20-2Z, 40-4Z, 60-9Z, BC00-0Z, 20,40-4Z, 60-9Z, BD10-1Z; ICD-10 I05-09, I30-I52;
5A) Arrhythmia: ICD-11 BC60-9Z; ICD-10 I44-49
5B) Heart Failure (HF): ICD-11 BD10-1Z; ICD-10 I50;
5C) Valvular Heart Disease (VHD): ICD-11 BB60-9Z, BC00-0Z; ICD-10 I05-08, I34-38;

Supplementary Material S5. NOS assessment of the included observational studies.

**Study**	Selection	Comparability	Outcome	Scores
Liu et al., 2013	3	1	2	6
Cuirong et al., 2024	3	1	2	6
Bade et al., 2019	3	1	2	6
Bray et al., 2023	3	2	2	7
Canıvar et al., 2017	3	1	2	6
Chan et al., 2023	3	1	2	6
Clarson et al., 2020	3	2	2	7
Dalleywater et al., 2015	3	2	1	6
Fisher et al., 2019	3	1	2	6
García et al., 2011	4	2	1	7
Hubbard et al., 2008	4	2	2	8
Khor et al., 2024	3	1	1	5
Kilpeläinen et al., 2023	3	1	1	5
Kato et al., 2021	3	1	2	6
Kim et al., 2015	4	2	2	8
Kizilirmak et al., 2023	3	2	1	6
López et al., 2023	3	1	1	5
Margaritopoulos et al., 2024	3	1	2	6
Miyake et al., 2005	3	2	2	7
Nathan et al., 2010	3	2	2	7
Nolan et al., 2022	3	2	1	6
Pedraza et al., 2018	2	2	1	5
Ponnuswamy et al., 2009	3	2	3	8
Sonaglioni et al., 2025	3	2	2	7
Sonaglioni et al., 2021	3	2	2	7
Sonaglioni et al., 2020	3	2	2	7
Sun et al., 2023	3	1	2	6
Yalniz et al., 2019	3	1	1	5

Supplementary Material S6. CV endpoint definitions and pooled effect estimates (95% CI).

Study	CV endpoint	Effect Measure (OR/RR)	Value (95% CI)	Note
Liu et al(13), 2013	PH	OR	0.23 (0.04, 1.14)	Data statistics for Overall CV Disease
Cuirong et al(14), 2024	CAD	OR	2.12 (1.32, 3.39)	
	PH	OR	1.90 (1.17, 3.10)	
	Arrhythmia	OR	1.04 (0.45, 2.38)	
Bade et al(15), 2019	CAD	OR	2.18 (1.63, 2.93)	
	PTE	OR	1.75 (1.11, 2.76)	
	PH	OR	11.80 (4.34, 32.10)	
Bray et al(16), 2023	CAD	OR	3.50 (2.16, 5.68)	Data statistics for Overall CV Disease, controls with COPD
	CAD	OR	14.74 (6.62, 32.79)	Data statistics for Overall CV Disease, for healthy control group
Canıvar et al(17), 2017	HF	OR	4.06 (0.19, 87.38)	
	IHD	OR	1.32 (0.29, 5.98)	
Chan et al(18), 2023	IHD	OR	3.13 (1.33, 7.37)	
	AF	OR	1.00 (0.37, 2.73)	
	PH	OR	1.05 (0.46, 2.40)	
Clarson et al(19), 2020	IHD (MI)	RR	1.75 (1.59, 1.93)	Data statistics for Overall CV Disease (cohort)
Dalleywater et al(20), 2015	IHD	RR	1.09 (0.91, 1.32)	Data statistics for Overall CV Disease (cohort)
Fisher et al(21), 2019	MI	OR	2.61 (1.60, 4.26)	Data statistics for Overall CV Disease
García et al(22), 2011	Unspecified CV diseases	OR	2.16 (1.01, 4.63)	Data statistics for Overall CV Disease
Hubbard et al(23), 2008	IHD (acute)	RR	1.53 (1.08, 2.15)	Data statistics for Overall CV Disease (cohort)
	IHD (acute)	OR	1.76 (1.47, 2.10)	
	AF	OR	1.34 (0.97, 1.84)	
	DVT	OR	1.97 (1.13, 3.44)	
Khor et al(24), 2024	Unspecified CV diseases	OR	2.42 (2.02, 2.90)	Data statistics for Overall CV Disease
Kilpeläinen et al(25), 2023	Unspecified CV diseases	OR	1.65 (0.97, 2.80)	Data statistics for Overall CV Disease
Kato et al(26), 2021	CAD	OR	2.22 (1.43, 3.45)	
	HF	OR	2.29 (1.43, 3.66)	

Kim et al(27), 2015	Acute IHD	OR	2.18 (1.41, 3.35)	Data statistics for Overall CV Disease
	Acute IHD	RR	1.46 (0.85, 2.50)	Data statistics for Overall CV Disease (cohort)
Kizilirmak et al(28), 2023	Unspecified CV diseases	OR	2.75 (0.65, 11.64)	Data statistics for Overall CV Disease
López et al(29), 2023	MI	OR	0.70 (0.28, 1.77)	
	HF	OR	1.37 (0.90, 2.07)	
	PH	OR	1.13 (0.89, 1.43)	
Margaritopoulos et al(30), 2024	PH	OR	1.94 (1.23, 3.04)	Data statistics for Overall CV Disease
Miyake et al(31), 2005	CAD	OR	2.02 (0.53, 7.65)	Data statistics for Overall CV Disease
Nathan et al(32), 2010	CAD	OR	2.22 (1.09, 4.52)	Data statistics for Overall CV Disease
Nolan et al(33), 2022	PH	OR	5.41 (1.53, 19.05)	Data statistics for Overall CV Disease
Pedraza et al(34), 2018	HF	OR	1.35 (1.26, 1.46)	
	VHD	OR	1.15 (1.04, 1.26)	
	PTE	OR	3.98 (3.56, 4.44)	
Ponnuswamy et al(35), 2009	AF	OR	1.12 (0.36, 3.55)	
	IHD	OR	2.67 (1.26, 5.64)	
Sonaglioni et al(36), 2025	CAS	OR	2.09 (1.10, 3.99)	
	CAD	OR	1.47 (0.35, 6.16)	
Sonaglioni et al(37), 2021	CAD	OR	1.59 (0.53, 4.78)	
	Arrhythmia	OR	2.02 (0.69, 5.87)	
Sonaglioni et al(38), 2020	CAD	OR	2.23 (1.28, 3.89)	
	VHD	OR	1.00 (0.32, 3.10)	
	Arrhythmia	OR	1.71 (0.42, 7.04)	
Sun et al(39), 2023	MI	OR	4.02 (1.81, 8.90)	
	AF	OR	1.81 (0.71, 4.63)	
	PTE	OR	3.57 (1.29, 9.90)	
	VTE	OR	0.94 (0.60, 1.48)	
Yalniz et al(40), 2019	CAD	OR	0.82 (0.22, 3.06)	Data statistics for Overall CV Disease

Studies reporting either overall risk outcomes (22,24,25,28) or only one specific outcome (13,16,19–21,23,27,30–33,40) were included for the overall CV disease risk assessment, as detailed in the note column (n=16, including 14 case-control/cross-sectional datasets and 4 cohort datasets). The same methodology was applied for subgroup analyses across different CVD categories and their specific subtypes.

Supplementary Material S7. Sensitivity analysis of CV disease (cohort studies).

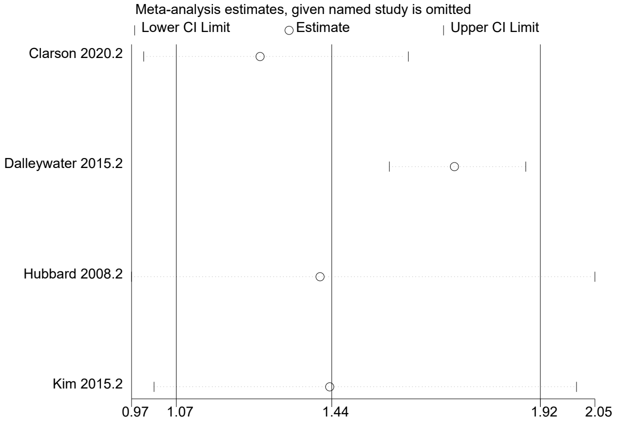

Supplementary Material S8. Sensitivity analysis of CV disease (case-control/cross-sectional studies). Every odds ratio was located between 1.84 and 3.24 while none of 95% confidence intervals crossed the invalid line ‘1’, signifying that the result was stable

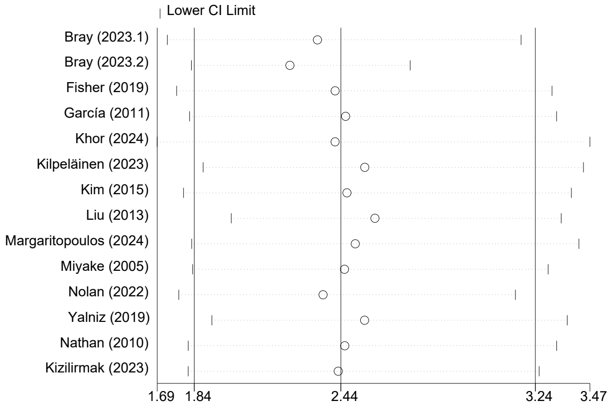

Supplementary Material S9. Meta-regression analyses of CV disease. (A) Based on control characteristic; (B) Based on data source; (C) Based on NOS score; (D) Based on certainty of CV disease diagnosis; (E) Based on regions; (F) Based on male%.

A

```
. metareg logOR Ctrl1 Ctrl2, wsse(_selogES) bsest(reml)

Meta-regression                                      Number of obs  =        14
REML estimate of between-study variance              tau2           =     .2874
% residual variation due to heterogeneity            I-squared_res  =    66.67%
Proportion of between-study variance explained       Adj R-squared  =    -2.08%
Joint test for all covariates                        Model F(2,11)  =      1.74
With Knapp-Hartung modification                      Prob > F       =    0.2203
```

logOR	Coefficient	Std. err.	t	P>|t|	[95% conf. interval]	
Ctrl1	-.2497965	.52711	-0.47	0.645	-1.409958	.9103647
Ctrl2	-.8337255	.483153	-1.73	0.112	-1.897138	.2296871
_cons	1.340578	.3918704	3.42	0.006	.4780773	2.203079

B

```
. metareg logOR datasource , wsse(_selogES) bsest(reml)

Meta-regression                                      Number of obs  =        14
REML estimate of between-study variance              tau2           =      .313
% residual variation due to heterogeneity            I-squared_res  =    66.84%
Proportion of between-study variance explained       Adj R-squared  =   -11.16%
With Knapp-Hartung modification
```

logOR	Coefficient	Std. err.	t	P>|t|	[95% conf. interval]	
datasource	.1971432	.4230838	0.47	0.650	-.7246773	1.118964
_cons	.7744422	.3075728	2.52	0.027	.1042987	1.444586

C

```
. metareg logOR NOS , wsse(_selogES) bsest(reml)

Meta-regression                                    Number of obs  =        14
REML estimate of between-study variance            tau2           =     .2359
% residual variation due to heterogeneity          I-squared_res  =    64.20%
Proportion of between-study variance explained     Adj R-squared  =    16.20%
With Knapp-Hartung modification
------------------------------------------------------------------------------
       logOR | Coefficient  Std. err.      t    P>|t|     [95% conf. interval]
-------------+----------------------------------------------------------------
         NOS |   .5421628   .3878356     1.40   0.187    -.3028583    1.387184
       _cons |   .6579418   .2512762     2.62   0.022     .1104579    1.205426
------------------------------------------------------------------------------
```

D

```
. metareg logOR diagnosis , wsse(_selogES) bsest(reml)

Meta-regression                                    Number of obs  =        14
REML estimate of between-study variance            tau2           =      .253
% residual variation due to heterogeneity          I-squared_res  =    65.50%
Proportion of between-study variance explained     Adj R-squared  =    10.14%
With Knapp-Hartung modification
------------------------------------------------------------------------------
       logOR | Coefficient  Std. err.      t    P>|t|     [95% conf. interval]
-------------+----------------------------------------------------------------
   diagnosis |   .4030307   .4003181     1.01   0.334    -.4691875    1.275249
       _cons |   .6941952   .2746948     2.53   0.027     .0956867    1.292704
------------------------------------------------------------------------------
```

E

```
. metareg logOR NorthAmerica Europe, wsse(_selogES) bsest(reml)

Meta-regression                                    Number of obs  =        14
REML estimate of between-study variance            tau2           =     .2167
% residual variation due to heterogeneity          I-squared_res  =    63.55%
Proportion of between-study variance explained     Adj R-squared  =    23.04%
Joint test for all covariates                      Model F(2,11)  =      2.31
With Knapp-Hartung modification                    Prob > F       =    0.1456
------------------------------------------------------------------------------
       logOR | Coefficient  Std. err.      t    P>|t|     [95% conf. interval]
-------------+----------------------------------------------------------------
NorthAmerica |   .7144343   .4245387     1.68   0.121    -.2199691    1.648838
      Europe |  -.1131864   .5045934    -0.22   0.827    -1.223789    .9974162
       _cons |   .5624063   .3389745     1.66   0.125    -.1836715    1.308484
------------------------------------------------------------------------------
```

F

```
. metareg logOR male, wsse(_selogES) bsest(reml)

Meta-regression                                    Number of obs  =        14
REML estimate of between-study variance            tau2           =     .3388
% residual variation due to heterogeneity          I-squared_res  =    68.03%
Proportion of between-study variance explained     Adj R-squared  =   -20.34%
With Knapp-Hartung modification
------------------------------------------------------------------------------
       logOR | Coefficient  Std. err.      t    P>|t|     [95% conf. interval]
-------------+----------------------------------------------------------------
        male |  -1.143394   1.856441    -0.62   0.549    -5.188232    2.901443
       _cons |   1.605753   1.202896     1.33   0.207    -1.015132    4.226639
------------------------------------------------------------------------------
```

Supplementary Material S10. Forest plots of different CV disease subgroup analyses. (A) Control Characteristic; (B) Data Source; (C) NOS score; (D) Certainty of CV Disease Diagnosis; (E) Groups matched by age; (F) BMI values matched; (G) Smoking status matched; (H) Diabetes prevalence matched.

A) Control Characteristic

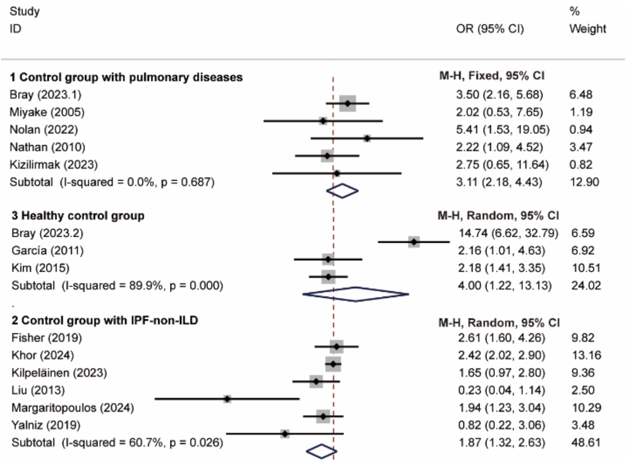

B) Data Source

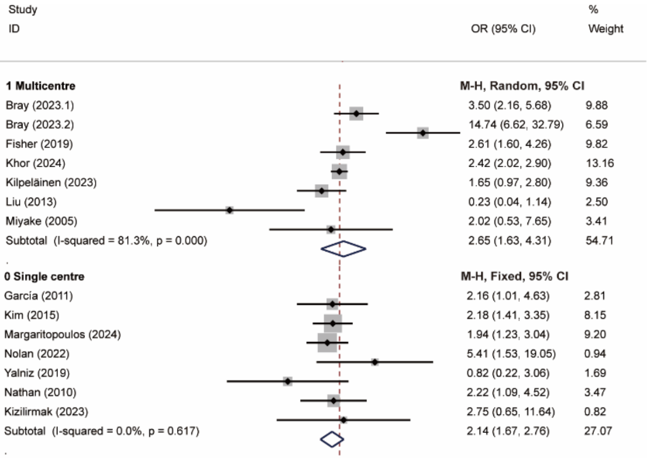

C) NOS score

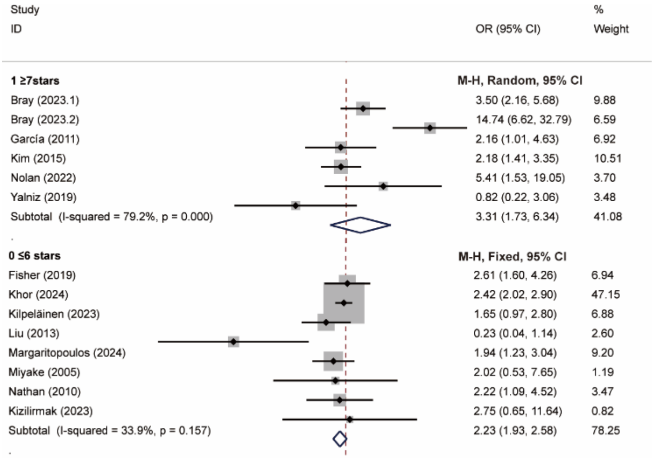

D) Certainty of CV Disease Diagnosis

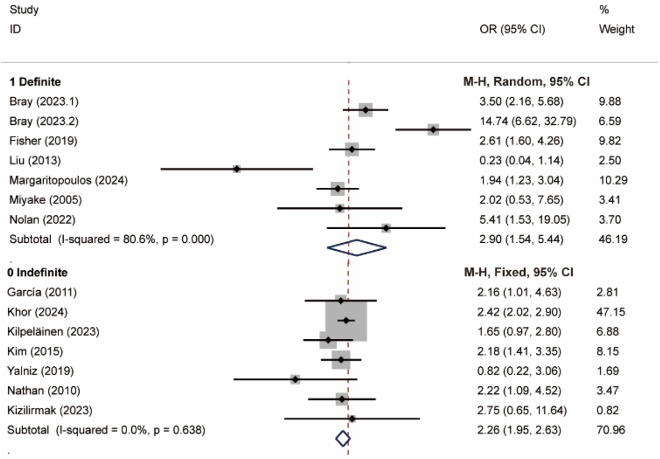

E) Groups matched by age

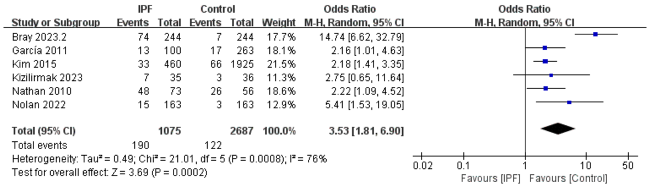

F) BMI values matched

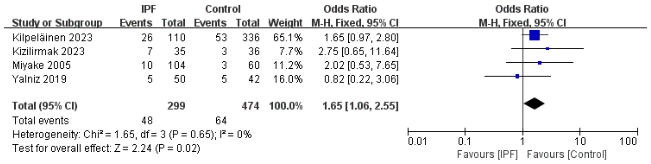

G) Smoking status matched

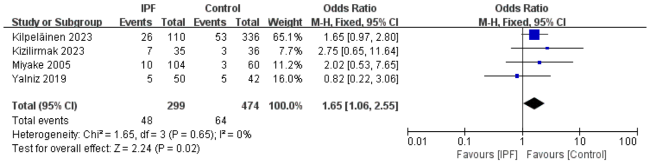

H) Diabetes prevalence matched

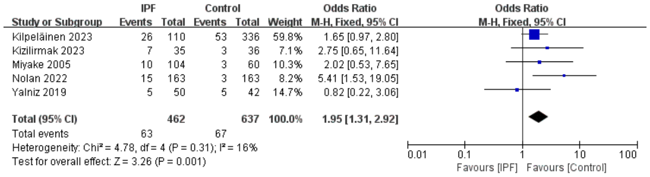

Supplementary Material S11. Forest plots of different CV disease categories. (A) IHD; (A1) Acute IHD; (A2) CAD; (B) Thromboembolic Disease; (C) PH; (D) Other Forms of Heart Disease; (D1) Arrhythmia; (D2) HF; (D3) VHD.

A

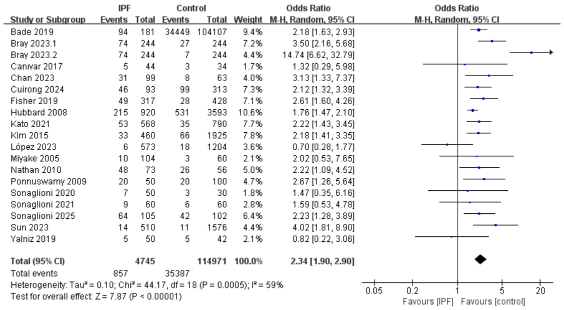

A1

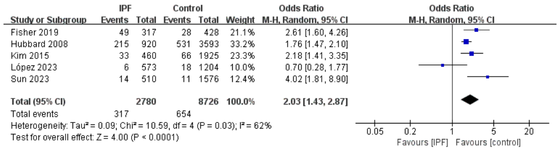

A2

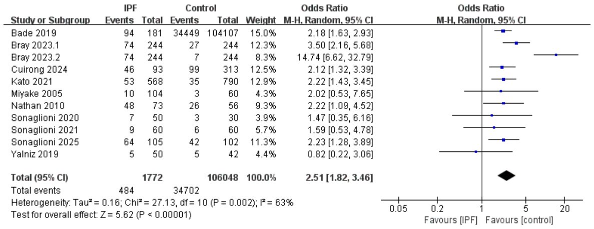

B

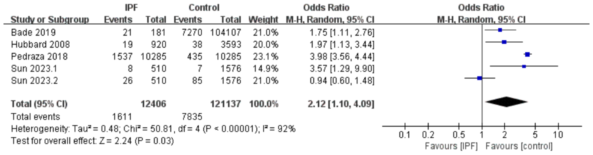

C

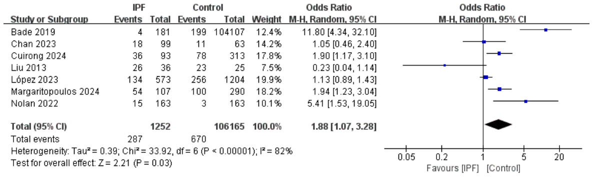

D

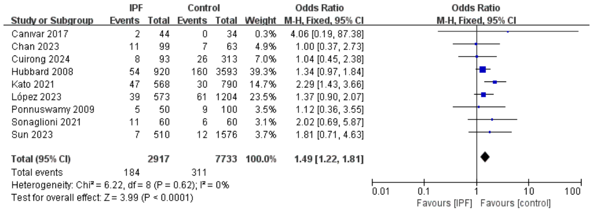

D1

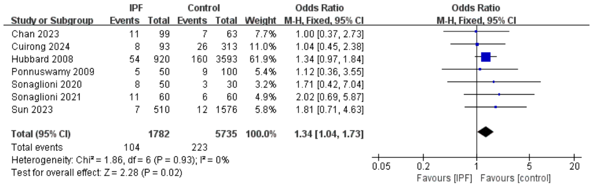

D2

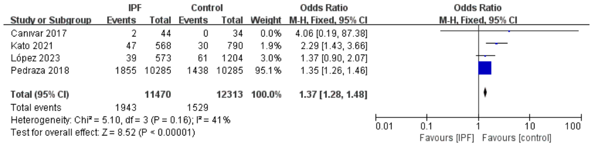

D3

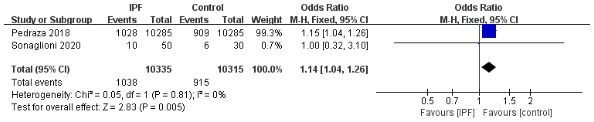

Supplementary Material S12. Funnel plots of CV diseases. (A) Overall CV Disease; (B) IHD; (B1) Acute IHD; (B2) CAD; (C) Thromboembolic Disease; (D) PH; (E) Other Forms of Heart Disease; (E1) Arrhythmia; (E2) HF; (E3) VHD.

A

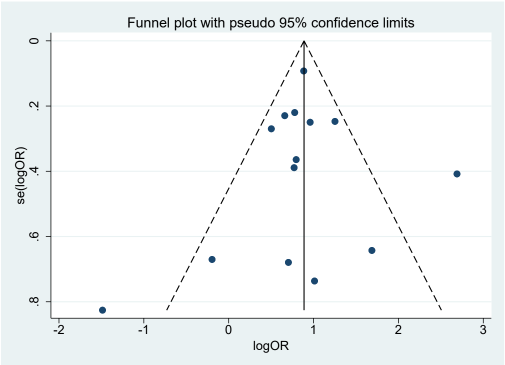

B

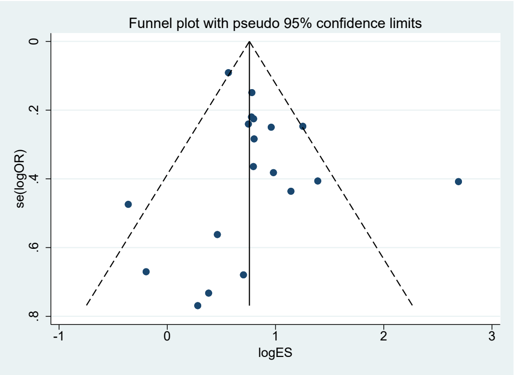

B1

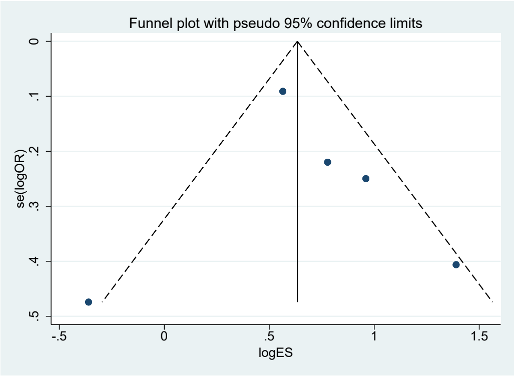

B2

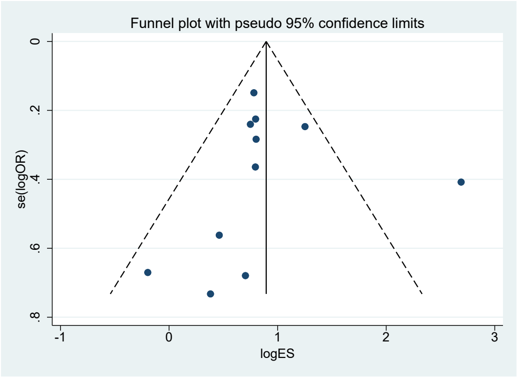

C

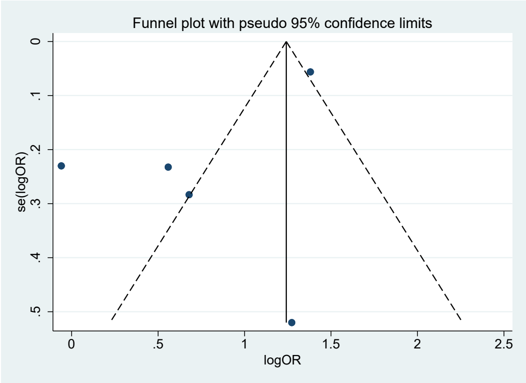

D

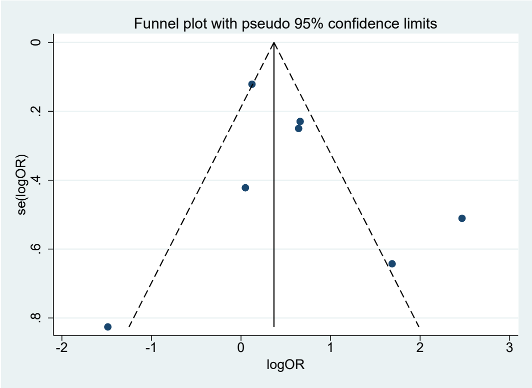

E

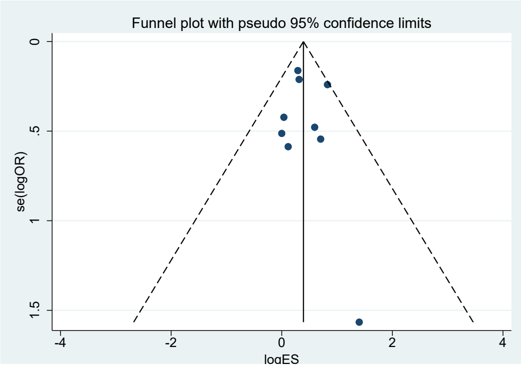

E1

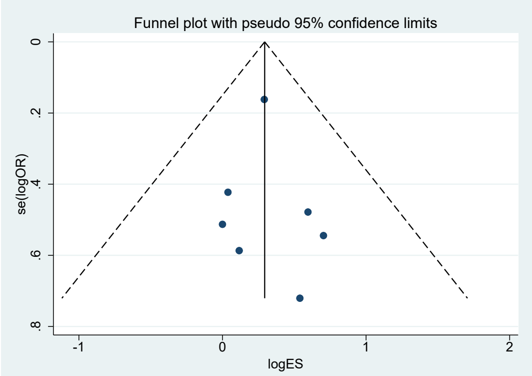

E2

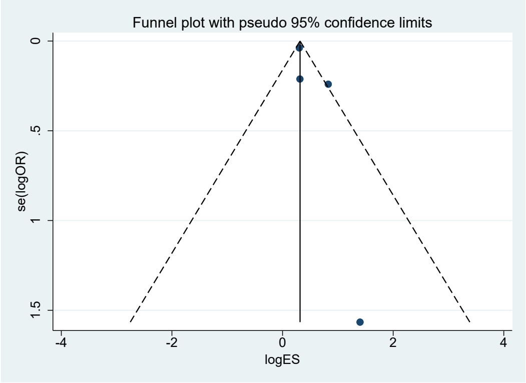

E3

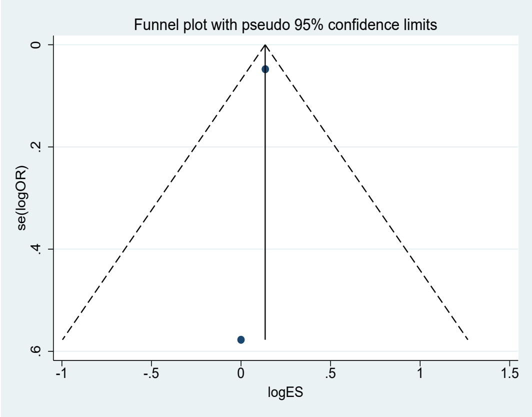

Supplement: Supplementary file 1 [file Data_Sheet_1.pdf]
